# PRAS40 prevents development of diabetic cardiomyopathy and improves hepatic insulin sensitivity in obesity

**DOI:** 10.1002/emmm.201303183

**Published:** 2013-10-31

**Authors:** Mirko Völkers, Shirin Doroudgar, Nathalie Nguyen, Mathias H Konstandin, Pearl Quijada, Shabana Din, Luis Ornelas, Donna J Thuerauf, Natalie Gude, Kilian Friedrich, Stephan Herzig, Christopher C Glembotski, Mark A Sussman

**Affiliations:** 1 SDSU Heart InstituteSan Diego, CA, USA; 2 Department of Biology, San Diego State UniversitySan Diego, CA, USA; 3 Joint Division Molecular Metabolic Control, DKFZ-ZMBH Alliance and Network Aging Research, German Cancer Research Center (DKFZ) Heidelberg, Center for Molecular Biology (ZMBH) and University Hospital, Heidelberg UniversityHeidelberg, Germany

**Keywords:** diabetes, PRAS40, mTOR

## Abstract

Diabetes is a multi-organ disease and diabetic cardiomyopathy can result in heart failure, which is a leading cause of morbidity and mortality in diabetic patients. In the liver, insulin resistance contributes to hyperglycaemia and hyperlipidaemia, which further worsens the metabolic profile. Defects in mTOR signalling are believed to contribute to metabolic dysfunctions in diabetic liver and hearts, but evidence is missing that mTOR activation is causal to the development of diabetic cardiomyopathy. This study shows that specific mTORC1 inhibition by PRAS40 prevents the development of diabetic cardiomyopathy. This phenotype was associated with improved metabolic function, blunted hypertrophic growth and preserved cardiac function. In addition PRAS40 treatment improves hepatic insulin sensitivity and reduces systemic hyperglycaemia in obese mice. Thus, unlike rapamycin, mTORC1 inhibition with PRAS40 improves metabolic profile in diabetic mice. These findings may open novel avenues for therapeutic strategies using PRAS40 directed against diabetic-related diseases.

## Introduction

The incidence and prevalence of type 2 diabetes mellitus (T2DM) are rising rapidly (Go *et al*, 2013). The World Health Organization has projected that diabetes related deaths will double between 2005 and 2030, and that T2DM will account for over 90% of all diagnosed diabetes in adults (http://www.who.int/diabetes/en/). Hyperglycaemia and hyperlipidaemia seen as a result of diabetes causes early maladaptation in cardiac energy metabolism with decreased glucose and increased fatty acid utilization, which is attributable to lipid accumulation, and toxicity in cardiomyocytes (Goldberg *et al*, 2012). Furthermore, selective hepatic insulin resistance is observed in patients with T2DM, where insulin fails to inhibit glucose production and maintains lipogenesis, contributing to, and exacerbating hyperglycaemia and hyperlipidaemia.

The important role of mechanistic target of rapamycin (mTOR) in maintaining tissue homeostasis is well documented. Both chronic activation or mTOR depletion are linked with defects in tissue function in a variety of organs including fat tissue, bone marrow and pancreas (Chen *et al*,^2008^; Gan *et al*, 2008; Kim & Chen, 2004; Rachdi *et al*, 2012). mTOR is also chronically elevated in nutrient overloaded obese mice and in humans (Laplante & Sabatini, 2012). mTOR senses inputs such as growth factors, nutrients and cellular energy status to regulate cellular growth, metabolism, and proliferation by both complex 1 (mTORC1) and complex 2 (mTORC2). Chronic increased mTORC1 activity causes insulin resistance through inhibition of the insulin receptor substrate 1 (IRS-1; Howell & Manning, 2011). However, previous studies with rapamycin to inhibit mTORC1 have generally failed to improve metabolic function in obesity-induced diabetes (Laplante & Sabatini, 2012). Given the defined roles of mTORC1 in highly metabolic organs like the heart or liver there is a need to delineate the pathophysiological role of deregulated mTORC1 signalling. An endogenous molecular mechanism exists that blocks mTORC1 activity to regulate growth by maintaining the appropriate balance between anabolic processes and catabolic processes. PRAS40 (proline rich Akt substrate of 40 kDa) is a specific component of mTORC1 that interacts with RAPTOR to inhibit mTORC1 kinase activity (Sancak *et al*, 2007; Vander Haar *et al*, 2007). PRAS40 was initially identified as a 14-3-3 binding protein (Kovacina *et al*, 2003) and was subsequently identified as an mTORC1 inhibitor and substrate (Sancak *et al*, 2007; Vander Haar *et al*, 2007; Oshiro *et al*, 2007). Results presented here demonstrate that mTORC1 inhibition with PRAS40 prevents the development of diabetic cardiomyopathy and improves hepatic insulin sensitivity, revealing a new target for treatment of T2DM and associated cardiomyopathy.

## Results

**Figure 1:**
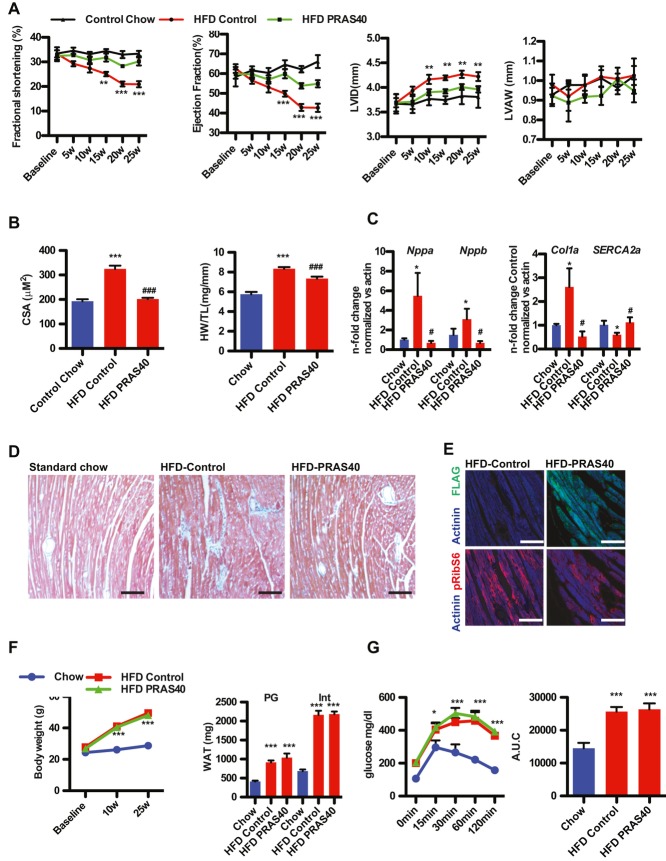
PRAS40 prevents HFD-induced cardiac dysfunction. A  Echocardiographic assessment of Chow-fed control or HFD fed mice for fractional shortening (FS), ejection fraction (EF) and end-diastolic dimension (LVID) and left anterior wall dimension (LVAW). **p < 0.03 *versus*control HFD, ***p < 0.008 *versus* control HFD. One-way anova *n* = 4 for Chow, *n* = 10 for HFD group. Values are mean ± SEM. B  Heart weight to tibia length ratio (HW/TL) in the indicated groups. (***p < 0.01 *vs*. control chow; ^###^*p* < 0.01 *vs*. control HFD). CSA in control and PRAS40 mice. ***p < 0.01 *versus* control chow; ^###^*p* < 0.05 *versus* control HFD. *n* = 4 for Chow, *n* = 10 for HFD group. One-way anova. Values are mean ± SEM. C  Nppa, Nppb. Col1 and SERCA2a gene expression. *p < 0.01 *versus* control chow; ^#^*p* < 0.05 *versus* control HFD. *n* = 4 for Chow, *n* = 10 for HFD group 1-way anova. Values are mean ± SEM. D  Masson-trichrome staining. HFD fed mice show distinct perivascular fibrosis. Bar = 150 μm. E  Representative confocal scans for the Flag-tag and actinin (green and blue, top panel) and pRibS6 and actinin (blue and red, bottom panel) in the indicated groups Bar = 30 μm. F  Body weight and white adipose tissue (WAT) weight. *n* = 4 for Chow, *n* = 10 for HFD group ***p < 0.01 *versus* control chow. One-way anova. Values are mean ± SEM. G  Glucose tolerance test and Area under the curve (A.U.C.) in Chow-fed control mice, and AAV-control or AAV-PRAS40 mice fed HFD for 25 weeks. *n* = 4 for Chow, *n* = 5 for HFD group ***p < 0.01 *versus* control chow. One-way anova. Values are mean ± SEM.

mTORC1 inhibition by PRAS40 was confirmed in cultured isolated neonatal myocytes (NRCM) as evidenced by decreased phosphorylation of S6Kinase (S6K) and blunted increase in cell size in following stimulation with high serum or fatty acids (supplementary Fig 1A–D). The effects of mTORC1 inhibition by PRAS40 were tested in a model of T2DM induced by high fat diet (HFD). Selective mTORC1 inhibition in cardiomyocytes *in vivo* was achieved using PRAS40 delivered via recombinant cardiotropic adeno-associated vector serotype 9 (AAV9) driven by a cardiomyocyte-specific myosin light chain (MLC2v) promoter construct (supplementary Fig 1E). Increased mTORC1 activity was observed in mice on a HFD (supplementary Fig 1F). In addition, increased PRAS40 protein levels in diabetic hearts were observed (supplementary Fig 1D). AAV-PRAS40 or AAV-control was injected at 7 weeks of age and mice were fed HFD chow of or standard for an additional 25 weeks. Baseline measurements were identical among the standard chow-fed groups, therefore were presented as a single control group. Diabetic cardiomyopathy is characterized by left ventricular dysfunction, and significant changes in the structure of the heart independent of coronary artery disease (Boudina & Abel, 2007). Decreased cardiac function was observed in the HFD control group after 15 weeks measured by echocardiography, but preserved in the HFD PRAS40 group (Fig 1A). This preservation of function was associated with decreased left ventricular diastolic dimension (LVID) and improved diastolic function (supplementary Table 3). Pathological growth of cardiomyocytes is a hallmark of failing myocardium. Increase in cell size was completely blunted by PRAS40, which was accompanied by a decrease in the HW/TL ratio (Fig 1B). *Nppa* and *Nppb* transcription were increased after HFD, indicative of hypertrophic growth, but blocked in AAV-PRAS40 mice (Fig 1C). In contrast, SERCA2a expression were decreased after HFD, indicative of cellular remodelling, but unchanged in AAV-PRAS40 mice. Collagen 1 expression increases after HFD and increased perivascular fibrosis was observed after HFD, but PRAS40 blocks cellular remodelling after HFD (Fig 1C and D). Decreased RibS6 phosphorylation was observed in paraffin-embedded sections form the HFD PRAS40 group compared to HFD control group (Fig 1E). Increases in body weight, white adipose tissue weight and size of adipocytes were identical between AAV-PRAS40 and AAV-control (Fig 1F, supplementary Fig 2A). Similarly, decrease in glucose tolerance was similar between the groups, confirming that cardiomyocyte-specific overexpression of PRAS40 did not alter the systemic effects of HFD (Fig 1G). In addition, tail vein injection of AAV9-PRAS40 did not increase PRAS40 levels in other tissues (supplementary Fig 2B and C). Similar results were obtained in the *db*/*db* mouse, which exhibits key characteristics of T2DM, namely hyperinsulinaemia, insulin resistance, hyperglycaemia, and develops diabetic cardiomyopathy with decreased cardiac function (Battiprolu *et al*, 2012). Myocardial dysfunction was prevented in AAV-PRAS40 treated *db*/*db* mice compared to the AAV-control group, as seen by significantly higher cardiac ejection fraction (EF%), fractional shortening (FS%) measured by serial echocardiography, associated with blunted hypertrophic remodelling and improved insulin sensitivity (supplementary Fig 3A–D), without affecting body weight, glucose levels and liver pathology (supplementary Fig 3E and F). Blood insulin levels in db/db mice were increased, confirming that the mice still present hyperinsulinaemia during the experiment (supplementary Fig 3G). Thus, mTORC1 inhibition with PRAS40 in cardiomyocytes protects cardiac function and prevents cardiac remodelling during HFD-induced diabetes.

**Figure 2:**
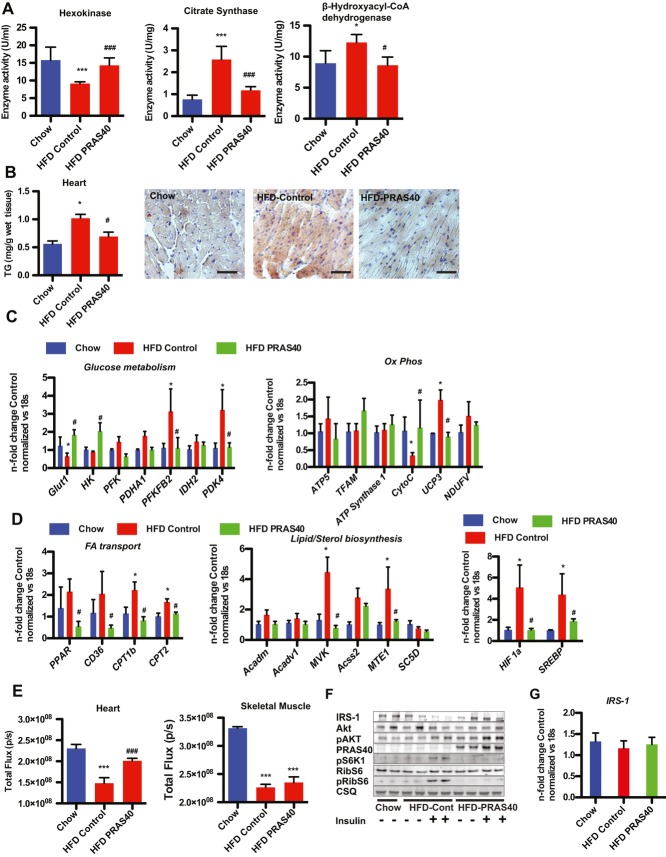
Metabolic remodelling induced by HFD is prevented by mTORC1 inhibition. A  Lower glycolytic (hexokinase) activity but increased oxidative (citrate synthase and β-hydroxy-acyl-CoA dehydrogenase) in HFD-fed AAV controls compared to AAV-PRAS40 mouse hearts. ***p < 0.01 *versus*control chow; ^###^*p* < 0.01 *versus* control HFD. *n* = 4 for Chow, *n* = 5 for HFD group. One-way anova. Values are mean ± SEM. B  TG content in the heart *p < 0.01 *versus* control chow; ^#^*p* < 0.05 *versus*control HFD. *n* = 3 for Chow, *n* = 3 for HFD group. One-way anova. Values are mean ± SEM. Lipid accumulation evidenced by Oil Red O staining. Bar = 100 μm. C  The expression levels of genes from glucose metabolism and oxidatative phosphorylation were measured by qRT-PCR. *p < 0.05 *versus* control chow; ^#^*p* < 0.05 *versus* control HFD. *n* = 4 for Chow, *n* = 5 for HFD group. One-way anova. Values are mean ± SEM. D  The expression levels of genes from FA metabolism and lipid and sterol biosynthesis were measured by qRT-PCR. Expression levels of HIF1a and SREBP *p < 0.05 *versus* control chow; ^#^*p* < 0.05 *versus* control HFD. *n* = 4 for Chow, *n* = 5 for HFD group. One-way anova. Values are mean ± SEM. E  Glucose uptake, visualized by fluorescence imaging of Xenolight RediJect 2-DG 750 in cardiac and skeletal muscle. ***p < 0.01 *versus* control chow; ^###^p < 0.01 *versus* control HFD. *n* = 4 for Chow, *n* = 4 for HFD group. One-way anova. Values are mean ± SEM. F  Immunoblots of proteins involved in insulin signalling. G  IRS-1 gene expression levels in the indicated groups.

**Figure 3:**
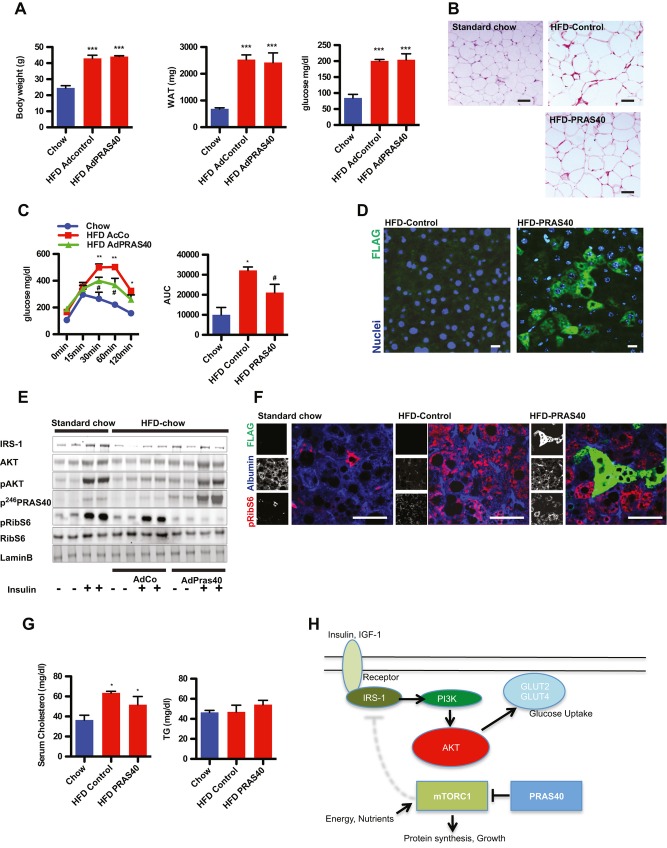
Hepatic insulin sensitivity is improved by PRAS40. A  Body weight, white adipose tissue (WAT) weight and baseline glucose levels in Chow-fed control mice, and Ad-control or Ad-PRAS40 mice fed HFD for 10 weeks. ***p < 0.01 *versus* control chow. *n* = 4 for Chow, *n* = 6 for HFD group. One-way anova. Values are mean ± SEM. B  H&E staining of WAT bar = 50 μm. C  Glucose tolerance test and area under the curve (AUC) in Chow-fed control mice, and Ad-control or Ad-PRAS40 mice fed HFD for 10 weeks. *p < 0.01*versus* control chow; ^#^*p* < 0.01 *versus* control HFD. *n* = 4 for Chow, *n* = 6 for HFD group. One-way anova. Values are mean ± SEM. D  Paraffin-embedded sections from control livers and PRAS40 treated liver stained for FLAG-tag (green) and nuclei (blue). Bar = 10 μm. E  Immunoblots of proteins involved in insulin and mTORC1 signalling. F  Paraffin-embedded sections from control livers and PRAS40 treated liver stained for pRibS6 (red), FLAG (green) and albumin (blue). Bar = 50 μm. G  Serum cholesterol and triglycerides levels in the indicated groups *p < 0.01*versus* control. *n* = 4 for Chow, *n* = 4 for HFD group. One-way anova. Values are mean ± SEM. H  mTORC1 signalling attenuates AKT activation through feedback mechanisms. PRAS40 blocks mTORC1 activation, improves insulin sensitivity, resulting in decreased growth and improved metabolic function.

T2DM causes an increase in FA utilization and a decrease in glycolytic oxidation in cardiomyocytes. Hexokinase activity was significantly decreased in HFD-fed AAV-control mouse hearts, but preserved in PRAS40 treated mice whereas citrate synthase activity and β-hydroxy-acyl-CoA dehydrogenase activity were increased in HFD-fed mice and this increase was blunted in AAV-PRAS40 hearts (Fig 2A). Increased FA supply and uptake results in lipid accumulation in diabetic cardiomyocytes, which is known to contribute to diabetic cardiomyopathy (Battiprolu *et al*, 2012). Triglyceride content in AAV-control and AAV PRAS40 mice was increased in the liver of HFD-fed mice, but was significantly lower in AAV-PRAS40 hearts compared to control (Fig 2B, supplementary Fig 4A). In addition, lipid accumulation in cardiomyocytes evidenced by Oil Red O staining was decreased in AAV-PRAS40 hearts, but not in liver sections (Fig 3B, supplementary Fig 4B).

Transcription profiling confirmed a decrease in expression of glycolytic genes such as hexokinase 2 and Glut1, and a decrease in cytochrome C expression in HFD-fed control hearts and an increase in the expression of PDK4, which is known to shift the cells from glycolysis to FA utilization, and this change was prevented by PRAS40 (Fig 2C). In contrast, expression of genes involved in FA transport, or lipid/sterol biosynthesis were increased in HFD-fed control hearts, but remained unchanged in PRAS40 hearts (Fig 2D). mTORC1 activation drives gene expression of SREBP (Düvel *et al*, 2010). SREBP expression increased in HFD-fed control hearts, but remained unchanged in PRAS40 hearts. To support the functional consequence of this, myocardial glucose uptake was measured using XenoLight RediJect 2-DeoxyGlucosone visualized with an *in vivo* imaging system. Glucose uptake in the myocardium after insulin stimulation was significantly lower in HFD-fed control mice compared to chow-fed mice, but significantly higher in AAV-PRAS40 hearts, whereas uptake into the skeletal muscle was impaired in both AAV-control and AAV-PRAS40 HFD groups, confirming that AAV-PRAS40 specifically targets cardiomyocytes (Fig 2E). Mechanistically, alterations in insulin signalling were prevented in AAV-PRAS40 hearts. IRS-1 expression was decreased in HFD-fed AAV-controls, but unchanged in AAV-PRAS40, and AKT phosphorylation was increased, indicating that mTORC1 inhibition with PRAS40 prevents insulin resistance in myocytes (Fig 2F). IRS-1 mRNA levels were unchanged, suggesting that the regulation of IRS-1 expression is regulated at the protein level (Fig 2G). mTORC1 inhibition by PRAS40 was confirmed by decreased phosphorylation of RibS6. The *in vivo* findings were confirmed in isolated myocytes. IRS-1 expression was increased, but IRS-1 phosphorylation decreased consistent with increased Akt^S473^ and Akt^T308^ phosphorylation (supplementary Fig 5A) after PRAS40 overexpression. Glucose uptake was increased by PRAS40 overexpression and associated with increased phosphorylation of AS160, an Akt downstream target involved in translocation of the glucose transporter Glut4 to the plasma membrane. Increased glucose uptake after PRAS40 overexpression was blocked by pharmacological inhibition of Akt, suggesting that Akt signalling improvement explain beneficial effects of PRAS40 (supplementary Fig 5B and C). Together, these data indicate that mTORC1 inhibition with PRAS40 prevents cardiac metabolic remodelling in response to HFD by preventing insulin resistance. mTORC1 inhibition by PRAS40 prevents cell-autonomous changes in cardiomyocytes that are responsible for the heart dysfunction in diabetic cardiomyopathy.

Hepatic insulin resistance is a major problem in the pathogenesis of T2DM. Impairment of Akt signalling in the liver promotes gluconeogenesis and contributes to hyperglycaemia observed in insulin resistance and T2DM. Therefore, the hypothesis was tested that mTORC1 inhibition with PRAS40 in the liver improves hepatic glucose metabolism and insulin sensitivity. Mice were fed HFD-chow for 10 weeks and were injected with either a control adenovirus (AdCo) or adenovirus encoding PRAS40 (AdPRAS40). Body weight and weight of the intestinal white adipose were unchanged after short-term PRAS40 overexpression in the liver (Fig 3A). Individual cell size of white apidocytes was identical between HFD-fed groups (Fig 3B). Strikingly, the response of AdPRAS40 injected mice challenged with glucose was significantly improved (Fig 3C). Expression of PRAS40 in hepatocytes was confirmed by immunohistochemistry with staining for the FLAG-tag (Fig 3D). To test the insulin response, insulin was injected into control and PRAS40 animals that were fasted for 6 h, and livers were collected after injection. Increased Akt^S473^phosphorylation was markedly reduced in HFD-fed control animals but largely restored after AdPRAS40 injection (Fig 3E). IRS-1 expression was normalized in PRAS40 livers compared to controls consistent with our data in the heart and phosphorylation of RibS6 was decreased, consistent with decreased mTORC1 activity by PRAS40. mTORC1 inhibition by PRAS40 in hepatocytes was confirmed in paraffin sections from livers stained against phosphorylated RibS6 protein (Fig 3F). Adverse effects of systemic rapamycin include hyperlipidaemia, but serum cholesterol and triglyceride levels were unchanged after liver-directed PRAS40 therapy (Fig 3G). These data confirm that short-term inhibition of mTORC1 with PRAS40 improves hepatic glucose metabolism and reduces hepatic insulin resistance, probably by improving IRS-1-Akt signalling (Fig 3H).

## Discussion

The increasing prevalence of obesity and associated metabolic disorders are major challenges for our society. T2DM is characterized by hyperglycaemia with insulin resistance as a cardinal feature manifesting together with obesity in many, but not all clinical cases. The involvement of mTORC1 activation in the pathogenesis of insulin resistance and T2DM has previously been studied (Howell & Manning, 2011; Wullschleger *et al*, 2006), but direct evidence that mTORC1 activation is causal for the pathogenesis of diabetic cardiomyopathy is missing. The results presented in this report show that inhibition of mTOR signalling and ensuing fat accumulation in cardiomyocytes can prevent metabolic remodelling and cardiac dysfunction. PRAS40 overexpression prevents cardiac dysfunction, despite the presence of chronic systemic metabolic dysfunction, suggesting that cell-autonomous changes in cardiomyocytes are responsible for the heart dysfunction in diabetic cardiomyopathy, which is in line with a previous report in drosophila (Birse *et al*, 2010).

Studies with rapamycin to inhibit mTORC1 have generally failed to improve metabolic function in obesity-induced diabetes (Laplante & Sabatini,^2012^). Although a recent study suggested that duration of rapamycin treatment has differential effects on metabolism in mice (Fang *et al*,^2013^), studies in patients showed that systemic administration of rapamycin worsened glucose and lipid status (Stallone *et al*, 2009). The systemic effects of rapamycin render it difficult to identify the tissue(s) that are responsible for beneficial or detrimental effects. Cell type specific mTORC1 inhibition with PRAS40, an endogenous mTORC1 component, is therefore a novel approach to prevent the deleterious consequences of chronic mTORC1 activation in obesity. AAV9 gene therapy with PRAS40 protected against diabetic cardiomyopathy. PRAS40 also improved liver sensitivity to insulin, suggesting that the phenotype observed in the myocardium is not cell type specific and could be translated into other important organs involved in the pathogenesis of T2DM, *i.e*. the pancreas. Moreover, a double PRAS40 therapy in liver and heart tissue might result in synergistic therapeutic effects.

Enhanced hepatic lipid synthesis leads to an increase in circulating FAs and TGs that in turn further increase FA transport into cardiomyocytes. Consequences of the increase FAs uptake are FAs accumulation in the cell and lipotoxicity when the oxidative capacity of the myocytes is exceeded. PRAS40 prevents the switch to enhanced FA oxidation and improves insulin-dependent glucose metabolism. On a molecular level, this phenotype is associated with increased IRS-1 expression, and blunted hypertrophic pathological growth of cardiomyocytes, making PRAS40 a promising molecular target to prevent cellular and metabolic remodelling. However, mTORC1 activation is not only associated with pathological hypertrophy, but also with physiological hypertrophy, such as athlete's heart, and future studies are needed to delineate the relative role of mTORC1 activation during physiological hypertrophy.

Hepatic insulin resistance contributes to hyperglycaemia and hyperlipidaemia, which further promotes global insulin resistance. Overexpression of PRAS40 in hepatocytes improves global glucose metabolism and increases insulin sensitivity in hepatocytes. In summary, the results presented in this report demonstrate that chronic activation of mTORC1 is critical to the pathogenic consequences of T2DM. Reducing mTORC1 activity by PRAS40 prevents cardiac dysfunction as well as cellular and metabolic remodelling in cardiomyocytes. This is not cell type specific as overexpression in the liver improves insulin sensitivity, suggesting that correctly timed inhibition of mTORC1 with PRAS40 is a feasible approach to target the diabetic consequences on a cellular level.

## Materials and Methods

### Mice and cardiac function analysis

All experiments were performed in 7-week-old male C57BL/6 and *db*/*db* (leptin receptor mutant) mice that were purchased from Jackson Laboratories. Starting at 8 weeks of age C57BL/6 mice were fed a HFD (60% fat—Research diets—Diet#: D12492) for 25 weeks, or shorter as indicated in the results. Controls were fed a standard chow diet for the same duration. Tail vein injection of either AAV-control or AAV-PRAS40 were performed at 7 weeks of age. Adenoviruses encoding GFP or PRAS40 were given to mice at the dose of 2 × 10^9^ virus particles by injection into the tail vein. Glucose tolerance tests were performed after fasting overnight. For echocardiography, mice were anaesthetized with 2% isoflurane and scanned using a Vevo770 imaging system (Visual Sonics, ON, Canada), as previously described (Quijada *et al*, 2012). For *in vivo* injection of insulin, mice were injected i.p. with PBS or insulin (1 U/kg/BW for 1 h) after overnight starvation. Sixteen-hour-fasted mice underwent GTT by i.p. injection with 1 g glucose per kg of BW. Blood glucose levels were measured at 0, 15, 30, 60 and 120 min with a Bayer Contour Glucometer. Institutional Animal Care and Use Committee approval was obtained for all animal studies.

### Isolation and primary cultures of neonatal ventricular cardiomyocytes

Isolation and primary cultures of neonatal and adult ventricular cardiomyocytes were prepared by standard procedures (Völkers *et al*, 2011). Cells were treated with serum (10%) or fatty acids supplements (F7050) as indicated in figures. A pharmacological Akt Inhibitor (Akt V—Calbiochem 124012-1MG) was used (10 μM) to block Akt activity. Recombinant adenoviruses were generated using human and mutated human PRAS40 cDNAs subcloned into the pShuttle-CMV vector using the AdEasy XL Adenoviral Vector system (Stratagene) as previously described (Muraski *et al*, 2007). The human full-length of PRAS40 contained a C-terminal FLAG tag. NRCMS were infected with adenoviruses as described previously at a multiplicity of infection of 20 (Cheng *et al*, 2011; Most *et al*, 2004).

### Adeno-associated virus serotype 9 (AAV9) generation and systemic *in vivo* AAV9 cardiac-targeted gene transfer protocol

*In vivo* cardiac-targeted PRAS40 expression in normal mouse hearts was obtained by using tail vein injection of an adeno-associated virus serotype 9 (AAV9) harbouring the PRAS40 driven by a cardiomyocyte-specific CMV-MLC2v0.8 promoter. Recombinant AAV9 vector carrying the same promoter without a downstream encoded transgene product served as control. Mice were anaesthetized with isoflurane (2%) and 100 μl of 37°C heated Ringer Lactate containing 1 × 10^11^ total viral particles of either AAV9-control, AAV9-PRAS40 or AAV9 shRictor were injected into the tail vein, as previously described (Völkers *et al*, 2011).

### Sample preparation, immunoblotting, RT-qPCR

Whole hearts and isolated myocytes were prepared as described previously using standard procedures (Völkers *et al*, 2011).

### Histology and staining

Sections for Masson's trichrome, H&E, and immunohistochemistry were generated from paraffin-embedded hearts. Sections for Oil Red O were generated from frozen sections. Hearts were perfused *in situ* with formalin for 15 min, excised, and fixed in formalin for 24 h at room temperature. Sections were deparaffinized using standard procedures. Immunostaining of paraffin-embedded hearts were performed, as described previously in detail (Avitabile *et al*, 2011).

### Enzyme activity, triglyceride content, blood cholesterol, triglyceride and insulin levels

Hexokinase enzyme activity was measured from heart lysates using the Hexokinase Colorimetric Assay Kit from BioVision according to the instructions. Citrate synthase activity and β-hydroxy-acyl-CoA dehydrogenase activity were measured using previous published protocols (Battiprolu *et al*, 2012). TG content in the liver and heart was analysed using the Triglyceride Colorimetric Assay Kit from Cayman Chemical according to the instruction. Blood levels of cholesterol, triglycerides and insulin were measured with commercially available ELISAs.

### Statistical analysis

Statistical analysis was performed using Student's *t*-test, and ANOVA as appropriate, with Tukey or Bonferroni *post hoc* tests. All data were analysed with GraphPad Prism 5.0 (Graphpad Software, Inc.); *p* values <0.05 were considered significant.

For more detailed Materials and Methods see the supplementary.

## Author contributions

MV and MAS performed study concept and design. MV, SD, NN, MHK, PQ, SD, LO, DJT, NG, KF, SH and CCG acquired data. MV, SD, KF, SH and MAS analysed and interpreted data. MV, CCG and MAS drafted the manuscript and performed critical revision. All the authors have approved the final version of the manuscript.
