# Supplementary material for: PRAS40 prevents development of diabetic cardiomyopathy and improves hepatic insulin sensitivity in obesity
Source: EMBO Mol Med. 2013 Oct 31;6(1):57–65. doi: 10.1002/emmm.201303183 (PMC3936489; doi:10.1002/emmm.201303183)
Supplement: Supplementary file 1 [file emmm0006-0057-sd1.pdf]

EMBO Molecular Medicine

PRAS40 prevents development of diabetic cardiomyopathy and improves hepatic insulin sensitivity in obesity

Mirko Volkers, Shirin Doroudgar, Nathalie Nguyen, Mathias H. Konstandin, Pearl Quijada, Shabana Din, Luis Ornelas, Donna J. Thuerauf, Natalie Gude, Kilian Friedrich, Christopher C. Glembotski and Mark A. Sussman

Corresponding author: Mark A. Sussman, San Diego State University

Review timeline:

Submission date:	14 June 2013
Editorial Decision:	15 July 2013
Revision received:	30 August 2013
Editorial Decision:	16 September 2013
Revision received:	23 September 2013
Accepted:	25 September 2013

Transaction Report:

(Note: With the exception of the correction of typographical or spelling errors that could be a source of ambiguity, letters and reports are not edited. The original formatting of letters and referee reports may not be reflected in this compilation.)

Editor: Céline Carret

1st Editorial Decision 15 July 2013

Thank you for the submission of your manuscript to EMBO Molecular Medicine. We have now heard back from the three referees whom we asked to evaluate your manuscript. Although the referees find the study to be novel and of potential interest, they also raise a number of concerns that need to be addressed in a major revision of this work.

As you will see from the comments below, all three referees acknowledge the novelty and medical relevance of the findings. However, all three also raise important points regarding the conclusiveness of the data and suggest performing additional experiments to better support the claims (adding more controls, increasing the mechanistic insight, clarifying the text, detailing the experimental protocols, etc) and validate the hypothesis.

In our view the suggested revisions would render the manuscript much more compelling and interesting to a broad readership. We therefore hope that you will be prepared to undertake the recommended experimental revision.

I look forward to receiving your revised manuscript.

***** Reviewer's comments *****

Referee #1 (Comments on Novelty/Model System):

The medical impact is medium based on the impossibility to use virus strategy to improve diabetic symptom today.

Referee #1 (Remarks):

In this study the authors examine the role of PRAS40, an inhibitor of the mTORC1 complex, on diabetic cardiomyopathy and hepatic insulin sensitivity using organ targeted virus strategy in mice. They show that inhibition of mTORC1 activity could be an option to prevent cardiac fat accumulation and heart dysfunction under high fat diet. The same strategy in liver enhances fatty acid oxidation and improves insulin sensitivity. Both result are linked with the loss of the known negative feedback of mTORC1 on the insulin signalling by IRS1 destabilization. This is the first study to examine in vivo the role of PRAS40 protein in the heart and liver. The authors suggest it as a future therapeutic strategy to target heart and liver symptom linked with diabetes. There are specific issues that need to be addressed in order to draw these conclusions:

1) The use of a db/db mice to show an hyperinsulinemia linked to cardiomiopathy is risky based on the loss of beta cell at 8 week. Does the author mean they use ob/ob mice? If not, confirmation of the blood insulin level must be done to show that the mice still present a hyperinsulinism.

2) Although the authors demonstrate that HFD decrease IRS1 expression but mTORC1 activity is linked with a loss of stability of IRS1. It is important to demonstrate whether this decrease occurs at the protein level, or at a RNA level.

3) The author seem to forget that rapamycin defect are linked with triacylglycerol lipolysis and release of free fatty acids and angiogenesis defect. In term of mTOR activation beneficial and detrimental effect on organ, the authors must mention the clear result described in adipocytes, hematopoietic and beta cell development (Kim JE & al, Diabetes 2004; Gan B & al, PNAS 2008; Chen C & al, J Exp Med 2008; Rachdi L & al, Diabetes 2012)

4) The authors describe hyperinsulinemia as a characteristic of T2DM. Are you sure? The authors describe obesity as a characteristic of T2DM. Is it always true?

5) In figure 1D, is it common to measure intestinal white adipose as an example of fat accumulation and not Perigonadal Fat Weight which is more easy to obtain? In figure 1 and 3, the authors should use 2 g/kg of glucose to challenge the metabolism of the mice and obtain significant data in their IPGTT. They are no significant data too in weight gain. How is it possible?

6) In figure 2F, P-rpS6 (RibS6) must be shown as a control of PRAS40 inhibition of mTORC1.

7) In figure 3E, P-rpS6 must be shown as a control of PRAS40 inhibition of mTORC1. In figure 3F, the authors should provide data regarding the cell types in which P-rpS6 is detected with a co-staining of Pras40 or the Flag. In figure 3G, the authors mention GLUT4 as a target without direct measurement. The glucose uptake is not in the liver only linked with GLUT4.

8) In figure S1, the scale bar is missing.

9) In figure S3, the gain in term of phosphorylation of akt in Serine 308 is not clear under Pras40 therapy.

10) What could be the result of a double Pras40 therapy in liver and in heart in the same time? Could we expect a synergic benefit?

Referee #2 (Comments on Novelty/Model System):

Technical quality is fine, but some experiments do not provide enough detail. Supplemental legends could also provide more detail.

The work is potentially highly novel and of medical relevance, but the work as presented does not go far enough to prove the hypothesis and would benefit from additional mechanistic insight.

Referee #2 (Remarks):

The authors provide intriguing data that overexpression of the mTORC1 inhibitor PRAS40 in the heart or liver rescues diabetic cardiomyopathy and liver insulin resistance. This work suggests that PRAS40 is a better therapeutic target than mTOR itself, as this strategy may avoid some of the adverse effects of systemic rapamycin treatment. The data that is shown is generally well done and clear. However, additional data would provide further mechanistic insight and help support the hypothesis.

Of major concern is that the central hypothesis is that HFD promotes excessive mTORC1 activity that drives the phenotype, and that PRAS40 rescue of this activity alleviates the phenotype. However, mTORC1 activity is not shown in the heart in response to HFD, nor is inhibition of this activity by PRAS40 overexpression shown. A rescue of phenotype is also shown in the db/db mouse, but these mice are presumably obese and diabetic due to hyperphagia on a chow diet due to absence of leptin signalling. How does the cardiac mTORC1 activity and PRAS40 compare to that induced by HFD in WT? Supplemental figure 1 shows inhibition of mTORC1 activity by PRAS40 overexpression in neonatal rat cardiomyocytes, but there is no stimulation and no HFD involved, so this does not address what is happening in the in vivo model. This data is provided for the liver study in Fig 3, but it would be important to show this for the heart. The authors state that "direct evidence that mTORC1 activation is causal for the pathogenesis of diabetic cardiomyopathy is missing" and claim that this study now provides this evidence. I would argue that they are close to being able to make this powerful conclusion, but without showing the mTORC1 data, it is not yet conclusively proven.

Because PRAS40 is both a substrate and inhibitor of mTORC1, this study raises the question of how PRAS40 expression and phosphorylation is affected by HFD or in db/db.

Work from the Manning lab shows that mTORC1 activity promotes gene expression related to glucose uptake, glycolysis, and fatty acid synthesis in mefs. The data in the current manuscript show a divergence, with PRAS40 overexpression inducing genes for glucose metabolism, but suppressing those for fatty acid synthesis. The Manning lab implicates HIF1a and SREBP in this gene expression. Are these genes affected by HFD and PRAS40 in the heart? This could add additional mechanistic insight.

While the echo data and Nppa/b data is consistent with diabetic cardiomyopathy, it would be helpful to also show some histological data including a trichrome stain to illustrate the heart phenotype and degree of rescue by PRAS40 at the cellular level in intact tissue. This would also provide a good opportunity to stain for phosphorylated ribosomal S6 as an index of mTORC1 activity. Additional qPCR markers could also be included, such as SERCA2a as an index of heart failure.

It should perhaps be noted in the discussion that mTORC1 activity is also associated with physiological hypertrophy, such as athlete's heart.

Adverse effects of systemic rapamycin include impaired glucose metabolism and hyperlipidemia. Does liver-directed PRAS40 affect this?

Minor points:
Fig 2F It would be helpful to have the insulin control in the Chow group.
The phosphorylation site(s) in the anti-pIRS1 antibody should be specified.
There are multiple inconsistencies/typos in figure numbers in the text and figure legends. Supp Fig 3E is mentioned in the legend but not shown in the figure. Bottom of page 6 refers to Fig 3B but should be 2B. Bottom of page 7 refers to 2E, should be 2F.

Referee #3 (Comments on Novelty/Model System):

This is the first report addressing a relevant role for mTOR activation in the development of diabetic cardiomyopathy. The authors used an original approach to constrain mTOR activity, by inhibiting mTORC1 overexpressing the proline rich Akt substrate 40 (PRAS40). By using this strategy, the authors demonstrate that PRAS40-dependent inhibition of mTORC1 ameliorate the metabolic profile of diabetic cells and mice.
The experiments are well performed, enough clear also to non-specialists, and adequate cell and animal models have been used. This reviewer suggests only one major experiment to improve this work and have some minor comments.

Referee #3 (Remarks):

Volkers et al., describe a new role for mTORC1 inhibition by PRAS40 in preventing the development of diabetic cardiomyopathy and in ameliorating cardiac and hepatic metabolic response in a mouse model of type 2 diabetes mellitus (T2DM), suggesting IRS-1/Akt signalling as a major determinant in these processes. According to this reviewer, the results shown in this paper demonstrate a surprising effect of PRAS40 in blunting the detrimental consequences of cardiac maladaptation to T2DM. The experiments are technically well performed and suggest new therapeutic approaches to overcome and prevent the onset of diabetic cardiomyopathy as well as to ameliorate hepatic metabolism.
This reviewer has only one additional experiment to suggest and some minor comments regarding this paper.

Major comments.
1. The authors suggest that IRS-1/AKT signalling improvement probably explains the beneficial effects of PRAS40-dependent mTORC1 inhibition on hepatic glucose metabolism (Results section, page 9, lines 1-3). Basically the same results were found in isolated myocytes (Results section, page 7, lines 19-23 and page 8, lines 1-5, supporting figure 5). If so, the inhibition of Akt phosphorylation (by means of commercially available inhibitors), in the presence of overexpressed PRAS40, should revert PRAS40 ability to preserve hepatic glucose metabolism and cardiac functions. This reviewer is conscious that performing these inhibition experiments in vivo is time consuming, therefore suggests to perform at least the in vitro experiments shown in supporting fig. 1A, B and 5A in the presence or absence of an Akt inhibitor to validate this hypothesis.
Minor comments.
1. Results section, page 6 line 23 and page 7 line 2: the results described are related to figure 2B not to figure 3B.
2. Supporting figure 5 is not divided into panels. The authors should erase "A" above the western blot results and refer to figure 5, not ot figure 5A, in the MS.
3. Page 15, Impact section: in this paragraph, two different fonts are used.
4. Page 11, lines 17-18: PRAS40 reduces mTORC1 activity, not the protein level.
5. Figure 3E, Supporting Figure 3D: please indicate PRAS40 phosphorylated residue (is it Thr246?), as this protein may be phosphorylated at many sites (11 phosphopeptides have been detected, Wang et al., JBC, 2007).

1st Revision - authors' response 30 August 2013

Reviewer #1 Comments: **In this study the authors examine the role of PRAS40, an inhibitor of the mTORC1 complex, on diabetic cardiomyopathy and hepatic insulin sensitivity using organ targeted virus strategy in mice. They show that inhibition of mTORC1 activity could be an option to prevent cardiac fat accumulation and heart dysfunction under high fat diet. The same strategy in liver enhances fatty acid oxidation and improves insulin sensitivity. Both results are linked with the loss of the known negative feedback of mTORC1 on the insulin signalling by IRS1 destabilization. This is the first study to examine in vivo the role of PRAS40 protein in the heart and liver. The authors suggest it as a future therapeutic strategy to target heart and liver symptom linked with diabetes. There are specific issues that need to be addressed in order to draw these conclusions:**

We appreciate the reviewer's assessment and have addressed the comments as follows:

<u>*Comments*</u>

1. ***The use of a db/db mice to show an hyperinsulinemia linked to cardiomiopathy is risky based on the loss of beta cell at 8 week. Does the author mean they use ob/ob mice? If not, confirmation of the blood insulin level must be done to show that the mice still present a hyperinsulinism.***

This is an important point and we agree that we need to confirm hyperinsulinemia in the mice. To address this important concern, additional data have been incorporated into the revised manuscript. We measured blood insulin levels in 8 week old db/db mice. Blood insulin levels were significantly increased in db/db mice compared to control animals, confirming that the db/db mice have hyperinsulimemia at the beginning of the experiment. These results are in line with published data from Dale Abel group (Endocrinology 146(12):5341–5349, Diabetes 60:1424–1434, 2011). These data are now included in the manuscript and mentioned in the text as delineated below.

Page 6 line 20:
Blood insulin levels in db/db mice were increased, confirming that the mice still present hyperinsulinemia during the experiment **(Supporting Figure 3G)**.

2. **Although the authors demonstrate that HFD decrease IRS1 expression but mTORC1 activity is linked with a loss of stability of IRS1. It is important to demonstrate whether this decrease occurs at the protein level, or at a RNA level.**

We appreciate the opportunity to address this excellent question and agree that it is important to understand how IRS-1 expression is regulated. We measured IRS-1 mRNA levels in heart samples from mice on control Chow diet or on HFD diet. IRS-1 mRNA levels were unchanged after HFD and PRAS40 did not affect IRS-1 mRNA levels; suggesting that regulation of IRS-1 expression is regulated at the protein level.

Page 8 line 10:
IRS-1 mRNA levels were unchanged, suggesting that the regulation of IRS-1 expression is regulated at the protein level (Figure 2G).

3. **The author seem to forget that rapamycin defect are linked with triacylglycerol lipolysis and release of free fatty acids and angiogenesis defect. In term of mTOR activation beneficial and detrimental effect on organ, the authors must mention the clear result described in adipocytes, hematopoietic and beta cell development (Kim JE & al, Diabetes 2004; Gan B & al, PNAS 2008; Chen C & al, J Exp Med 2008; Rachdi L & al, Diabetes 2012)**

We concur with the reviewer and mention the clear results of the role of mTOR in tissue homeostasis in the revised manuscript.

Page 3 line 12:
The important role of mechanistic target of rapamycin (mTOR) in maintaining tissue homeostasis is well documented. Both chronic activation or mTOR depletion are linked with defects in tissue function in a variety of organs including fat tissue, bone marrow and pancreas (Gan *et al*, 2008; Kim & Chen, 2004; Rachdi *et al*, 2012; Chen *et al*, 2008).

4. **The authors describe hyperinsulinemia as a characteristic of T2DM. Are you sure? The authors describe obesity as a characteristic of T2DM. Is it always true?**

The reviewer mentions an important point. We agree that hyperinsulinemia is not always a characteristic of T2DM, especially in the late phase of the disease. Moreover, T2DM can be found in non-obese patients. Accordingly, we changed the manuscript to clarify this important point.

Page 11 line 3:
T2DM is characterized by hyperglycemia with insulin resistance as a cardinal feature manifesting together with obesity in many, but not all clinical cases.

5. **In figure 1D, is it common to measure intestinal white adipose as an example of fat accumulation and not Perigonadal Fat Weight which is more easy to obtain? In figure 1 and 3, the authors should use 2 g/kg of glucose to challenge the metabolism of the mice and obtain significant data in their IPGTT. They are no significant data too in**

weight gain. How is it possible?
We added the Perigonadal Fat Weight data to the revised manuscript (see new Figure 1F) as requested by the reviewer.

Page 6 line 6:
Increases in body weight, white adipose tissue weight, and size of adipocytes were identical between AAV-PRAS40 and AAV-Control (**Figure 1F, Supporting Fig. 2A**).

We used 1g/Kg/BW of glucose for the glucose-tolerance test according to previously published protocols from different groups (Zechner et al, Cell Metabolism 12, 633–642, Mazumder et al, DIABETES, VOL. 53, SEPTEMBER 2004, Lamming et al, Aging Cell Aug:12 (4): 712-8). The glucose clearance after injection of 1g/kg glucose was significantly impaired after HFD, in line with previous reports using this dose (Zechner et al, Cell Metabolism 12, 633–642, Mazumder et al, DIABETES, VOL. 53, SEPTEMBER 2004, Lamming et al, Aging Cell Aug:12 (4): 712-8). We added the significance symbols to the revised Figure F-G. The increase in body weight was also significant and we added the significance levels to the revised Figure. We apologize for any confusion that we caused by forgetting to add the significance symbols and hope to have satisfactorily addressed the reviewers concerns.

6. **In figure 2F, P-rpS6 (RibS6) must be shown as a control of PRAS40 inhibition of mTORC1.**

Again, we agree with the reviewer that it is important to show mTORC1 inhibition by PRAS40. Additional immunoblots have been incorporated into the revised Figure 2F, which now shows robust inhibition of phosphorylated RibS6 in PRAS40 treated animals.

Page 8 line 12:
mTORC1 inhibtion by PRAS40 was confirmed by decreased phosphorylation of RibS6.

7. **In figure 3E, P-rpS6 must be shown as a control of PRAS40 inhibition of mTORC1. In figure 3F, the authors should provide data regarding the cell types in which P-rpS6 is detected with a co-staining of Pras40 or the Flag. In figure 3G, the authors mention GLUT4 as a target without direct measurement. The glucose uptake is not in the liver only linked with GLUT4.**

We added new immunoblots, which also show inhibition of phosphorylated RibS6 by PRAS40 in the liver (New Figure 3E). We also stained paraffin embedded liver sections with the hepatocyte cell marker albumin to determine the cell type in which PRAS40 and RibS6 are detected as requested by the reviewer. Albumin positive cells are also positive for phosphorylated RibS6 and PRAS40 positive cells (detected by the exogenous Flag-tag) clearly show less phosphorylated RibS6.

Page 9 line 18:
IRS-1 expression was normalized in PRAS40 livers compared to controls consistent with our data in the heart and phosphorylation of RibS6 was decreased, consistent with decreased mTORC1 activity by PRAS40.

Page 9 line 20:
mTORC1 inhibition by PRAS40 in hepatocytes was confirmed in paraffin sections from livers stained against phosphorylated RibS6 protein (**Figure 3F**)

We also agree with the reviewer that the glucose uptake in the liver is not only linked with GLUT4. We changed the schematic figure accordingly to avoid any confusion and also mention GLUT 2 as an important glucose transporter in the liver.

8. **In figure S1, the scale bar is missing.**

We apologize for the mistake and added the scale bar in Figure S1.

9. **In figure S3, the gain in term of phosphorylation of akt in Serine 308 is not clear under Pras40 therapy.**

We agree with the reviewer that it is important to quantify the phosphorylation of Akt in PRAS40 treated animals. Whereas phosphorylation at Ser^{473} was significantly increased the phosphorylation at Thr^{308} was unchanged in PRAS40 overexpressing hearts. This might be due to the fact that the

© EMBO

animals were fasted for 6h before the tissue was collected and therefore the IRS-1/PI3K signalling cascade was mostly inactive.

10. **What could be the result of a double Pras40 therapy in liver and in heart in the same time? Could we expect a synergic benefit?**

The reviewer asks an excellent question. It is tempting to speculate that a double PRAS40 therapy in the liver and in the heart would be synergistic by improving contractile function and hepatic insulin sensitivity and we hope to address this question in future on-going studies.

Page 12 line 2:
Moreover, a double PRAS40 therapy in liver and heart tissue might result in synergistic therapeutic effects.

We thank the reviewer for the erudite commentary and hope these revisions serve to appropriately address the provided critique.

Reviewer #2 Comments: **The authors provide intriguing data that overexpression of the mTORC1 inhibitor PRAS40 in the heart or liver rescues diabetic cardiomyopathy and liver insulin resistance. This work suggests that PRAS40 is a better therapeutic target than mTOR itself, as this strategy may avoid some of the adverse effects of systemic rapamycin treatment. The data that is shown is generally well done and clear. However, additional data would provide further mechanistic insight and help support the hypothesis.**
Of major concern is that the central hypothesis is that HFD promotes excessive mTORC1 activity that drives the phenotype, and that PRAS40 rescue of this activity alleviates the phenotype. However, mTORC1 activity is not shown in the heart in response to HFD, nor is inhibition of this activity by PRAS40 overexpression shown. A rescue of phenotype is also shown in the db/db mouse, but these mice are presumably obese and diabetic due to hyperphagia on a chow diet due to absence of leptin signalling.

We thank the reviewer for these supportive remarks and have addressed comments as follows:

1. **How does the cardiac mTORC1 activity and PRAS40 compare to that induced by HFD in WT?**

We concur with the reviewer that it is important to document the level of mTORC1 activity in after HFD compared to normal Chow diet. Additional immunoblots have been incorporated into the revised manuscript, which now show clear activation of mTORC1 in db/db mice and in mice on a HFD (new Supplemental Figure 1F). PRAS40 reduced mTORC1 activity in both db/db mice and after HFD (Supplemental Figure 3D and revised Figure 2F).

Page 5 line 10:
Increased mTORC1 activity was observed in mice on a high fat diet **(Supporting Fig. 1F)**.

Page 8 line 12:
mTORC1 inhibition by PRAS40 was confirmed by decreased phosphorylation of RibS6.

Page 9 line 18:
IRS-1 expression was normalized in PRAS40 livers compared to controls consistent with our data in the heart and phosphorylation of RibS6 was decreased, consistent with decreased mTORC1 activity by PRAS40.

2. **Supplemental figure 1 shows inhibition of mTORC1 activity by PRAS40 overexpression in neonatal rat cardiomyocytes, but there is no stimulation and no HFD involved, so this does not address what is happening in the in vivo model.**

This is another important point. To mimic more closely the *in vivo* situation of a fat rich HFD we performed additional experiments and we added Fatty acids (FA) to the media to activate mTORC1. FA stimulation caused a significant increase in cardiomyocyte size, which was associated with increased mTORC1 activation. This increase was blocked by PRAS40 overexpression. These data are now included in the manuscript and mentioned in the text as delineated below.

Page 5 line 2:
mTORC1 inhibition by PRAS40 was confirmed in cultured isolated neonatal myocytes (NRCM) as evidenced by decreased phosphorylation of S6Kinase (S6K) and blunted increase in cell size in following stimulation with high serum or fatty acids. (**Supporting Fig. 1A-D**).

3. **This data is provided for the liver study in Fig 3, but it would be important to show this for the heart. The authors state that "direct evidence that mTORC1 activation is causal for the pathogenesis of diabetic cardiomyopathy is missing" and claim that this study now provides this evidence. I would argue that they are close to being able to make this powerful conclusion, but without showing the mTORC1 data, it is not yet conclusively proven.**

See also point 1 and point 6 from reviewer#1. Additional data have been incorporated to show that first mTORC1 activity is increased in diabetic cardiomyopathy and second PRAS40 inhibits mTORC1 activation. These data are now included in the manuscript and mentioned in the text as delineated below.

Page 5 line 10:
Increased mTORC1 activity was observed in mice on a high fat diet **(Supporting Fig. 1F)**.

Page 8 line 12:
mTORC1 inhibtion by PRAS40 was confirmed by decreased phosphorylation of RibS6.

Page 6 line 4:
Decreased RibS6 phosphorylation was observed in paraffin embedded sections form the HFD PRAS40 group compared to HFD control group (**Figure 1E**).

4. **Because PRAS40 is both a substrate and inhibitor of mTORC1, this study raises the question of how PRAS40 expression and phosphorylation is affected by HFD or in db/db.**

We concur with the reviewer and additional immunoblots have been incorporated into the revised manuscript. PRAS40 protein expression increases both in db/db and after HFD, whereas the relative phosphorylation level of PRAS40 decreases. This might reflect a compensatory mechanism to inhibit sustained activation of mTORC1 in db/db mice and after HFD.

Page 5 line 10:
In addition, increased PRAS40 protein levels in diabetic hearts were observed **(Supporting Fig. 1F)**

5. **Work from the Manning lab shows that mTORC1 activity promotes gene expression related to glucose uptake, glycolysis, and fatty acid synthesis in mefs. The data in the current manuscript show a divergence, with PRAS40 overexpression inducing genes for glucose metabolism, but suppressing those for fatty acid synthesis. The Manning lab implicates HIF1a and SREBP in this gene expression. Are these genes affected by HFD and PRAS40 in the heart? This could add additional mechanistic insight.**

This is an excellent question and we measured HIF1a and SREBP gene expression in hearts after HFD. Interestingly, we found elevated HIF1a and SREBP gene expression after HFD, in line with the concept that mTORC1 drives gene expression of HIF1a and SREBP. This increase is blocked in PRAS40 expressing hearts, suggesting that increased mTORC1 is causal for the increase in gene expression. However, it is unknown if the increased expression of HIF1a or SREBP is causally involved in the pathogenesis of diabetic cardiomyopathy. These data are now included in the manuscript and mentioned in the text as delineated below.

Page 7 line 21:
mTORC1 activation drives gene expression of HIF1a and SREBP (Düvel *et al*, 2010). HIF1a and SREBP expression increased in HFD-fed control hearts, but remained unchanged in PRAS40 hearts.

6. **While the echo data and Nppa/b data is consistent with diabetic cardiomyopathy, it would be helpful to also show some histological data including a trichrome stain to illustrate the heart phenotype and degree of rescue by PRAS40 at the cellular level in**

intact tissue. This would also provide a good opportunity to stain for phosphorylated ribosomal S6 as an index of mTORC1 activity. Additional qPCR markers could also be included, such as SERCA2a as an index of heart failure.

We appreciate the opportunity to address this excellent question and agree that it is important to understand how PRAS40 confers protection at a cellular level in intact tissue. We performed Trichrome staining and HFD-fed control hearts show distinct perivascular fibrosis compared to the Chow fed group, and this perivascular fibrotic remodelling was blocked by PRAS40. In addition Collagen 1 expression was decreased by PRAS40 after HFD. These new data are now presented in the revised Figure 1C-D. Ribosomal S6 phosphorylation was increased in HFD-fed hearts, and this phosphorylation was inhibited by PRAS40, in line with our immunoblot analysis (see also point 3). The data are presented in the revised **Figure 1E**. SERCA2a expression declined in HFD-fed control heart compared to the Chow-fed group and this decrease was prevented by PRAS40 (New **Figure 1C**). The new data indicate together with the echo data that PRAS40 not only prevent cardiac dysfunction but also cellular remodelling in diabetic cardiomyopathy.

Page 6 line 1:

In contrast, SERCA2a expression were decreased after HFD, indicative of cellular remodelling, but unchanged in AAV-PRAS40 mice. Collagen 1 expression increases after HFD and increased perivascular fibrosis was observed after HFD, but PRAS40 blocks cellular remodelling after HFD (**Figure 1C-D**).

Page 6 line 4:

Decreased RibS6 phosphorylation was observed in paraffin embedded sections form the HFD PRAS40 group compared to HFD control group (**Figure 1E**).

7. **It should perhaps be noted in the discussion that mTORC1 activity is also associated with physiological hypertrophy, such as athlete's heart.**

We agree with the reviewer that mTORC1 activity is also increased during physiological hypertrophy. The discussion has been modified to address this important point.

Page 12 line 11:

However, mTORC1 activation is not only associated with pathological hypertrophy, but also with physiological hypertrophy, such as athlete’s heart, and future studies are needed to delineate the relative role of mTORC1 activation during physiological hypertrophy.

8. **Adverse effects of systemic rapamycin include impaired glucose metabolism and hyperlipidemia. Does liver-directed PRAS40 affect this?**

To address this question cholesterol and Triglyceride levels in the serum were analysed. Levels were unchanged after PRAS40 therapy (with a trend to decreased cholesterol levels after PRAS40 therapy).

Page 9 line 22:

Adverse effects of systemic rapamycin include hyperlipidemia, but serum cholesterol and Triglyceride levels were unchanged after liver-directed PRAS40 therapy (**Figure 3G**).

Minor points:

Fig 2F It would be helpful to have the insulin control in the Chow group.

We agree that this would be helpful to have insulin controls in the Chow group. Unfortunately, we don’t have aged matched mice that were treated with insulin after overnight fasting. However, we can show in Figure 3E that the insulin response in HFD-fed animals is impaired in the liver compared to the Chow group. We hope that the reviewer agrees that it is beyond the scope to repeat another experimental cohort, which would require another 25 weeks.

The phosphorylation site(s) in the anti-pIRS1 antibody should be specified.

We agree and specified the phosphorylation site ($Ser^{636/639}$)

There are multiple inconsistencies/typos in figure numbers in the text and figure legends. Supp Fig 3E is mentioned in the legend but not shown in the figure. Bottom of page 6 refers to Fig 3B but should be 2B. Bottom of page 7 refers to 2E, should be 2F.

We apologize for any inconsistencies/typos in the manuscript. We carefully revised the manuscript and we hope to correct the mistakes throughout the manuscript.

We are thankful for the constructive critique of reviewer #2 and hopeful that the articulated concerns have been satisfactorily redressed.

Reviewer #3 comments:

Volkers et al., describe a new role for mTORC1 inhibition by PRAS40 in preventing the development of diabetic cardiomyopathy and in ameliorating cardiac and hepatic metabolic response in a mouse model of type 2 diabetes mellitus (T2DM), suggesting IRS-1/Akt signalling as a major determinant in these processes. According to this reviewer, the results shown in this paper demonstrate a surprising effect of PRAS40 in blunting the detrimental consequences of cardiac maladaptation to T2DM. The experiments are technically well performed and suggest new therapeutic approaches to overcome and prevent the onset of diabetic cardiomyopathy as well as to ameliorate hepatic metabolism.

This reviewer has only one additional experiment to suggest and some minor comments regarding this paper.

We like to thank the reviewer very much for this positive evaluation.

1. **The authors suggest that IRS-1/AKT signalling improvement probably explains the beneficial effects of PRAS40-dependent mTORC1 inhibition on hepatic glucose metabolism (Results section, page 9, lines 1-3). Basically the same results were found in isolated myocytes (Results section, page 7, lines 19-23 and page 8, lines 1-5, supporting figure 5). If so, the inhibition of Akt phosphorylation (by means of commercially available inhibitors), in the presence of overexpressed PRAS40, should revert PRAS40 ability to preserve hepatic glucose metabolism and cardiac functions. This reviewer is conscious that performing these inhibition experiments in vivo is time consuming, therefore suggests to perform at least the in vitro experiments shown in supporting fig. 1A, B and 5A in the presence or absence of an Akt inhibitor to validate this hypothesis.**

We agree with the reviewer that it is important to document that IRS-1/AKT signalling improvement explains the beneficial effects of PRAS40-dependent mTORC1 inhibition. According to the suggestion of the reviewer we performed *in vitro* experiments in the presence or absence of an Akt inhibitor. Akt inhibition blocked the increase in Glucose uptake in isolated myocytes in PRAS40 overexpressing myocytes. Immunoblots confirmed successful inhibition of Akt using a commercially available inhibitor (AktV- Calbiochem). These data are now included in the manuscript and mentioned in the text as delineated below.

Page 8, line 19:

Increased Glucose Uptake after PRAS40 overexpression was blocked by pharmacological inhibition of Akt, suggesting that Akt signalling improvement explain beneficial effects of PRAS40 (**Supporting Figure 5B-C**).

Minor comments.

1. **Results section, page 6 line 23 and page 7 line 2: the results described are related to figure 2B not to figure 3B.**

We apologize for inconsistencies, and carefully revised the manuscript.

2. **Supporting figure 5 is not divided into panels. The authors should erase "A" above the western blot results and refer to figure 5, not to figure 5A, in the MS.**

The new Supporting Figure 5 is now divided into panels.

3. **Page 15, Impact section: in this paragraph, two different fonts are used.**

We revised the manuscript and only one font is used in the revised manuscript.

4. **Page 11, lines 17-18: PRAS40 reduces mTORC1 activity, not the protein level.**

We totally agree and changed the manuscript according to the reviewer’s suggestion.

© EMBO

Page 12, line 19:
Reducing mTORC1 activity by PRAS40 prevents cardiac dysfunction as well as cellular and metabolic remodelling in cardiomyocytes.

5. **Figure 3E, Supporting Figure 3D: please indicate PRAS40 phosphorylated residue (is it Thr246?), as this protein may be phosphorylated at many sites (11 phosphopeptides have been detected, Wang et al., JBC, 2007).**

We agree that PRAS40 is phosphorylated at many differed residues. We checked the PRAS40 phosphorylation at Thr^{246}, which is now indicated in the revised manuscript.

Thank you for fair and reasonable requests for manuscript revisions provided by the reviewers and we look forward to seeing their responses.

2nd Editorial Decision 16 September 2013

Thank you for the submission of your revised manuscript to EMBO Molecular Medicine. We have now received the enclosed reports from the referees that were asked to re-assess it. As you will see the reviewers are now supportive and I am pleased to inform you that we will be able to accept your manuscript pending the following final amendments:

- Please provide the missing scale bars as indicated by referee 1. We would also recommend to follow this referee's suggestion regarding comment 1 in order to avoid confusion.

I look forward to reading a new revised version of your manuscript as soon as possible.

***** Reviewer's comments *****

Referee #1 (Comments on Novelty/Model System):

The medical impact is medium based on the impossibility to use virus strategy to improve diabetic symptom today.

Referee #1 (Remarks):

The authors answer my question. Here is some last comments:

1. In Page 7 line 21, you write to answer one of your reviewers that "mTORC1 activation drives gene expression of HIF1a and SREBP (Düvel et al, 2010). HIF1a and SREBP expression increased in HFD-fed control hearts, but remained unchanged in PRAS40 hearts."
I think that Manning declare a controversial effect in his paper that he never discuss later. The only accepted link between mTORC1 and HIF1a expression is a higher translation of the mRNA producing enough protein of HIf1A that will let this transcription factor act on his target in normoxia. HFD generate some metabolism involving hypoxia that is not the purpose of mTORC1. I would advise to remove "HIF1a" and focus on SREBP in your sentences.
2. The scale bar is missing in Figure 1D-E, 2B, 3B suppl 2A, 4B.

Referee #2 (Comments on Novelty/Model System):

The study is carefully and thoroughly performed and provides novel mechanistic insights on the roles of mTORc1 and PRAS40 in the heart and liver complications of diabetes. The work has potential therapeutic implications.

Referee #2 (Remarks):

The authors have done a thorough and excellent job of responding to my criticisms, and those raised by the other reviewers. The manuscript has been strengthened by the new experiments and editing. The new data support and extend the conclusions and lend new mechanistic insights to their original observations.

Referee #3 (Comments on Novelty/Model System):

In the previous review, this reviewer positively evaluated this work. Now, the paper has been further improved by additional experiments, strengthening the authors' findings.

Referee #3 (Remarks):

The paper by Volkers et al. has been considerably improved. All this reviewer's concerns have been satisfactorily addressed. Therefore this reviewer feels this paper suitable for publication on EMM at this stage.

2nd Revision - authors' response 23 September 2013

Reviewer #1 Comments: **The authors answer my question. Here is some last comments:**
We appreciate the reviewer's assessment and have addressed the remaining comments as follows:

Comments

1. **In Page 7 line 21, you write to answer one of your reviewers that "mTORC1 activation drives gene expression of HIF1a and SREBP (Duvel et al, 2010). HIF1a and SREBP expression increased in HFD-fed control hearts, but remained unchanged in PRAS40 hearts." I think that Manning declare a controversial effect in his paper that he never discuss later. The only accepted link between mTORC1 and HIF1a expression is a higher translation of the mRNA producing enough protein of HIf1A that will let this transcription factor act on his target in normoxia. HFD generate some metabolism involving hypoxia that is not the purpose of mTORC1. I would advise to remove "HIF1a" and focus on SREBP in your sentences**.

We agree with the reviewer. We changed the manuscript according his advice and follow the recommendation of the handling editor.

Page 7, line 21
mTORC1 activation drives gene expression of SREBP (Düvel et al, 2010). SREBP expression increased in HFD-fed control hearts, but remained unchanged in PRAS40 hearts.

2. **The scale bar is missing in Figure 1D-E, 2B, 3B suppl 2A, 4B.**

We apologize and added the scale bar in each Figure in the revised manuscript

We thank the reviewer for the final commentary and hope these revisions serve to appropriately address the provided critique.

Reviewer #2 Comments: **The authors have done a thorough and excellent job of responding to my criticisms, and those raised by the other reviewers. The manuscript has been strengthened by the new experiments and editing. The new data support and extend the conclusions and lend new mechanistic insights to their original observations**.

We thank the reviewer for these supportive remarks.

Reviewer #3 comments:
The paper by Volkers et al. has been considerably improved. All this reviewer's concerns have been satisfactorily addressed. Therefore this reviewer feels this paper suitable for publication

on EMM at this stage.

We like to thank the reviewer very much for this positive evaluation.

Thank you for fair and reasonable requests for manuscript revisions provided by the reviewers.

© EMBO
