# Supplementary material for: PRAS40 prevents development of diabetic cardiomyopathy and improves hepatic insulin sensitivity in obesity
Source: EMBO Mol Med. 2013 Oct 31;6(1):57–65. doi: 10.1002/emmm.201303183 (PMC3936489; doi:10.1002/emmm.201303183)
Supplement: Supplementary file 2 [file emmm0006-0057-sd2.pdf]

Supporting Figure 1

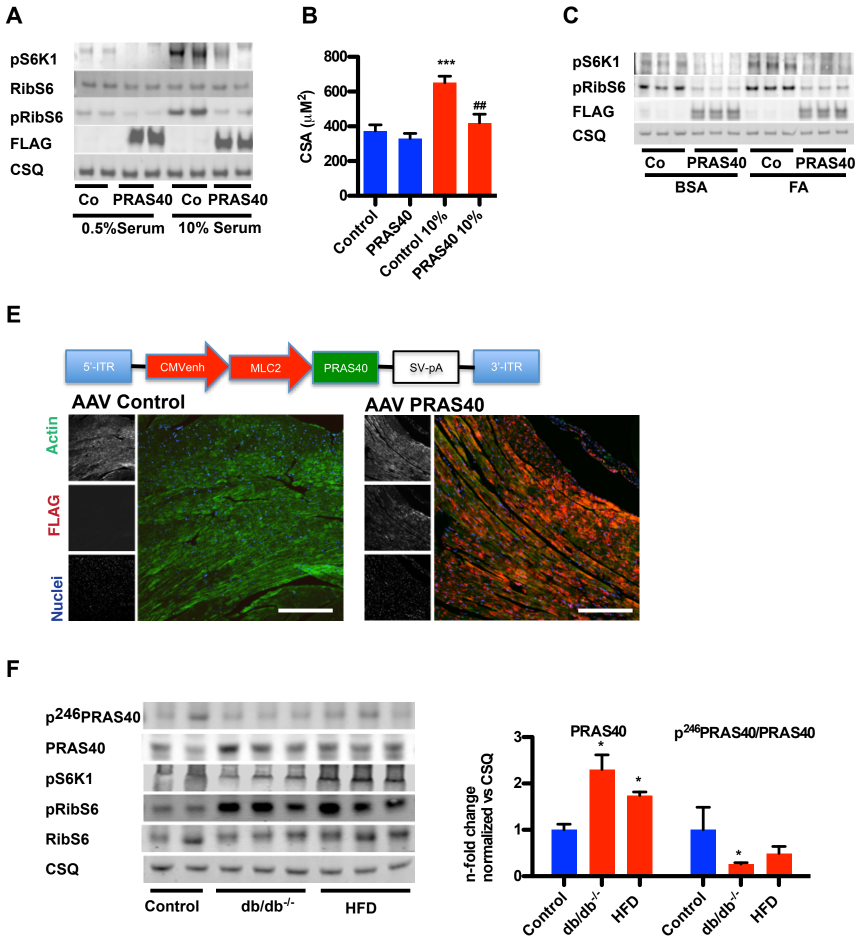

Supporting Figure 1. **(A)** mTORC1 activity is blocked by PRAS40 in response to high serum **(B)** Individual cell surface area (CSA) measurements from control or PRAS40 infected NRCMs and treated with 10%Serum (24h) (n=4 independent experiments, ***p<0.001 vs Control, ##p<0.01 vs Control 10%. **(C)** mTORC1 activity is blocked by PRAS40 in response to high Fatty acid concentrations (0.5ml/l) **(D)** Individual cell surface area (CSA) measurements from control or PRAS40 infected NRCMs and treated with Fatty Acids (24h) (n=3 independent experiments, *p<0.05 vs Control, ##p<0.01 vs Control FA. **(E)** Scheme of the AAV9PRAS40 under MLC2 promoter to overexpress PRAS40 in myocytes. Confocal microscopy of paraffin-embedded from PRAS40 mouse hearts stained for FLAG-tag (red), Actin (green) and nuclei (blue), scale bar 150μm. **(F)** Immunoblots showing mTORC1 and PRAS40 signaling in db/db and HFD mice. Barplots depicting quantitation of PRAS40 expression and p246/PRAS40 ratio . *p<0.05 versus control.
