# Supplementary material for: PRAS40 prevents development of diabetic cardiomyopathy and improves hepatic insulin sensitivity in obesity
Source: EMBO Mol Med. 2013 Oct 31;6(1):57–65. doi: 10.1002/emmm.201303183 (PMC3936489; doi:10.1002/emmm.201303183)
Supplement: Supplementary file 3 [file emmm0006-0057-sd3.pdf]

Supporting Figure 2

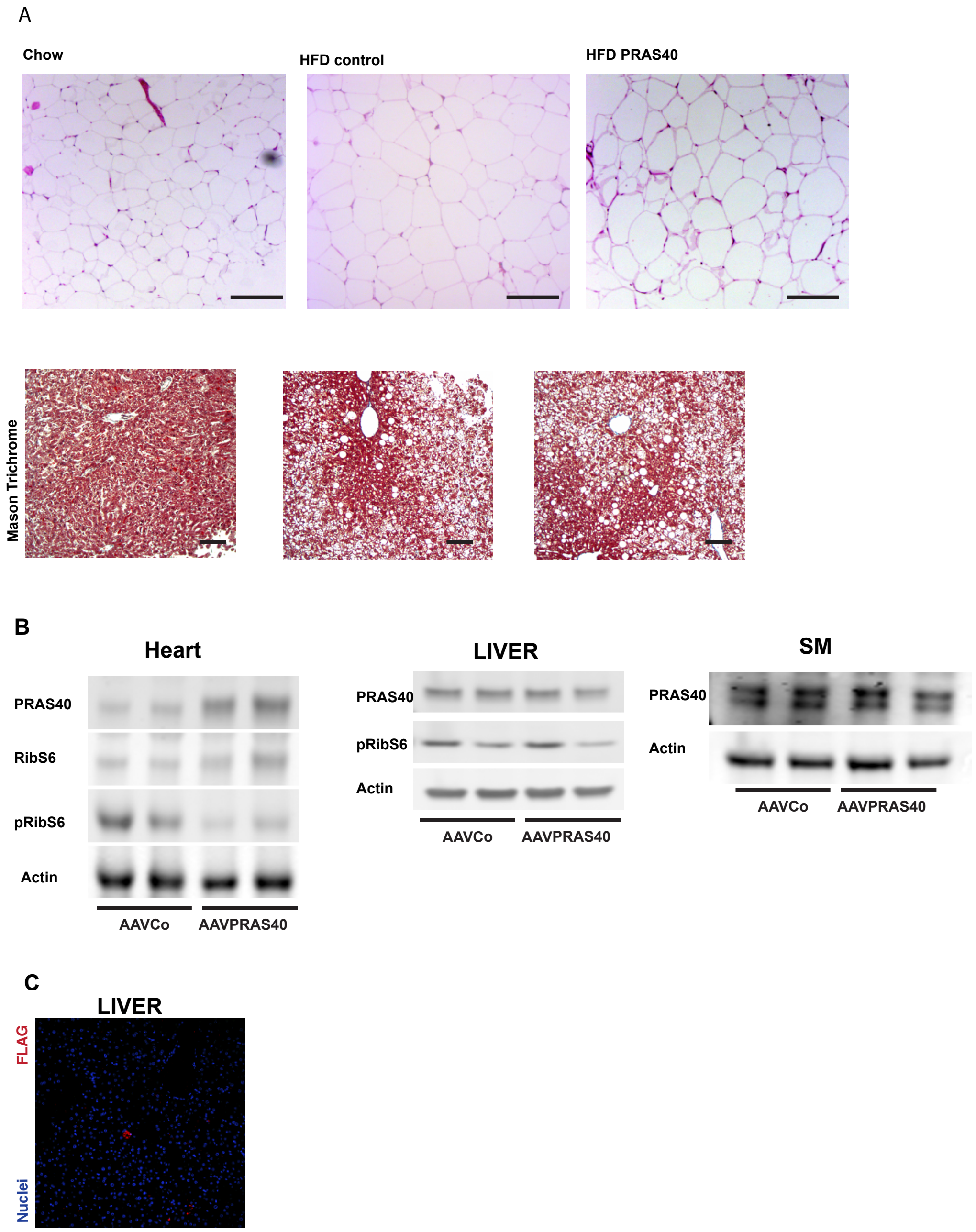

Supporting Figure 2. H&E staining of WAT sections from either Chow-fed or HFD-fed mice injected with AAV-Control or AAV-PRAS40. Mason-trichrome staining of liver sections from either Chow-fed or HFD-fed mice injected with AAV-Control or AAV-PRAS40. Scale bar 150μm. **(B)** Immunoblots from hearts, liver and skleletal muscle (SM) from either AAV-Control

or AAV-PRAS40 injected animals. **(C)** Confocal microscopy of paraffin-embedded liver sections after AAV-PRAS40 injections stained for FLAG-tag (red), Actin (green) and nuclei (blue). Only single cells are positive for the FLAG-tag.

Supporting Figure 3

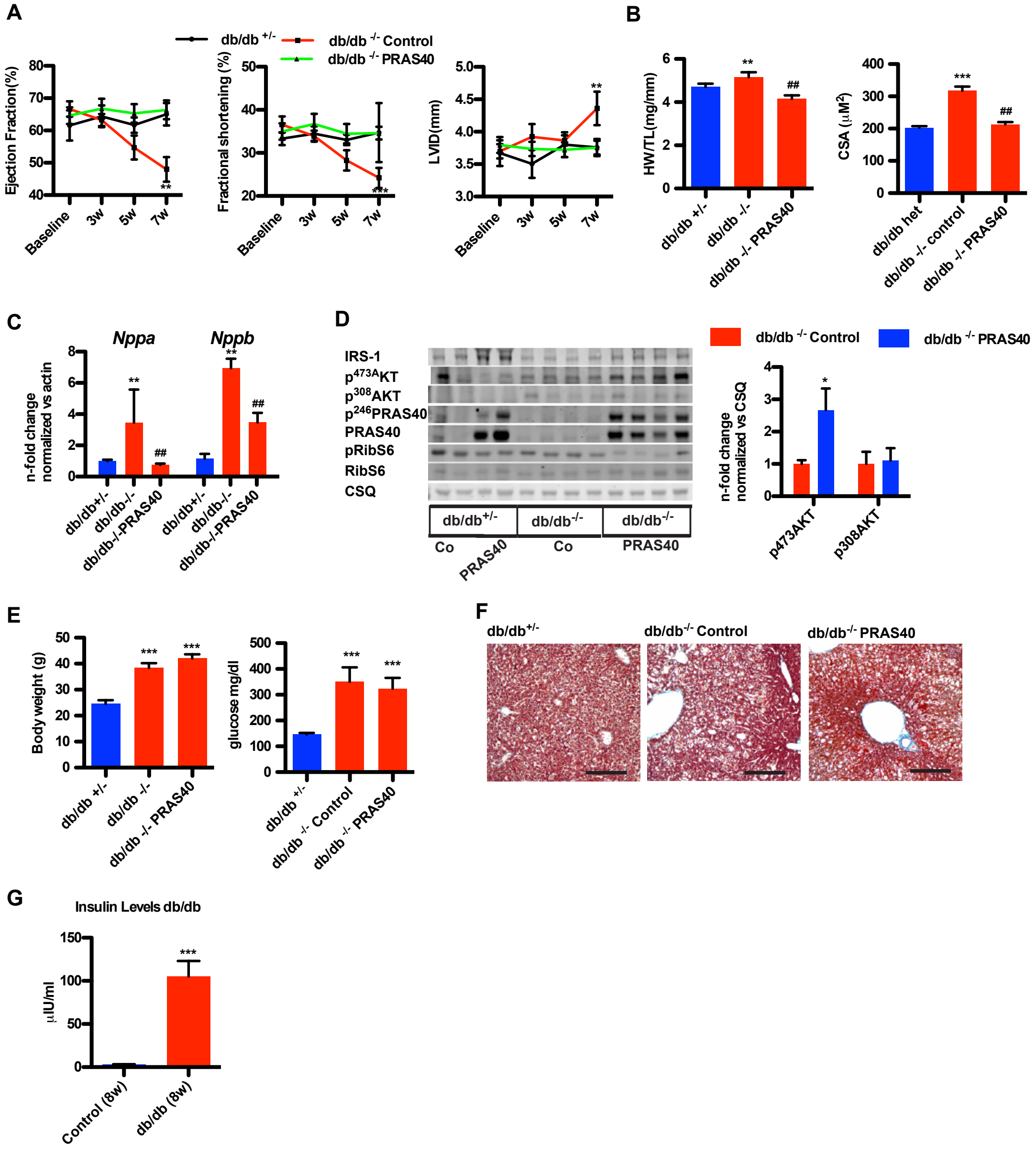

Supporting Figure 3. PRAS40 prevents diabetic cardiomyopathy in db/db mice. **(A)** Line graphs representing echocardiographic assessment of heterozygous control or *db/db* mice for percentage of fractional shortening (FS), ejection fraction (EF) and end-diastolic dimension (LVID), **p<0.01 vs control *db/db*. n=4 per het and 6 per *db/db* group. **(B)** Heart weight to tibia length ratio (HW/TL) in the indicated groups.. (**p<0.01 versus control het; ##p<0.01 versus
