# Supplementary material for: PRAS40 prevents development of diabetic cardiomyopathy and improves hepatic insulin sensitivity in obesity
Source: EMBO Mol Med. 2013 Oct 31;6(1):57–65. doi: 10.1002/emmm.201303183 (PMC3936489; doi:10.1002/emmm.201303183)
Supplement: Supplementary file 4 [file emmm0006-0057-sd4.pdf]

or AAV-PRAS40 injected animals. **(C)** Confocal microscopy of paraffin-embedded liver sections after AAV-PRAS40 injections stained for FLAG-tag (red), Actin (green) and nuclei (blue). Only single cells are positive for the FLAG-tag.

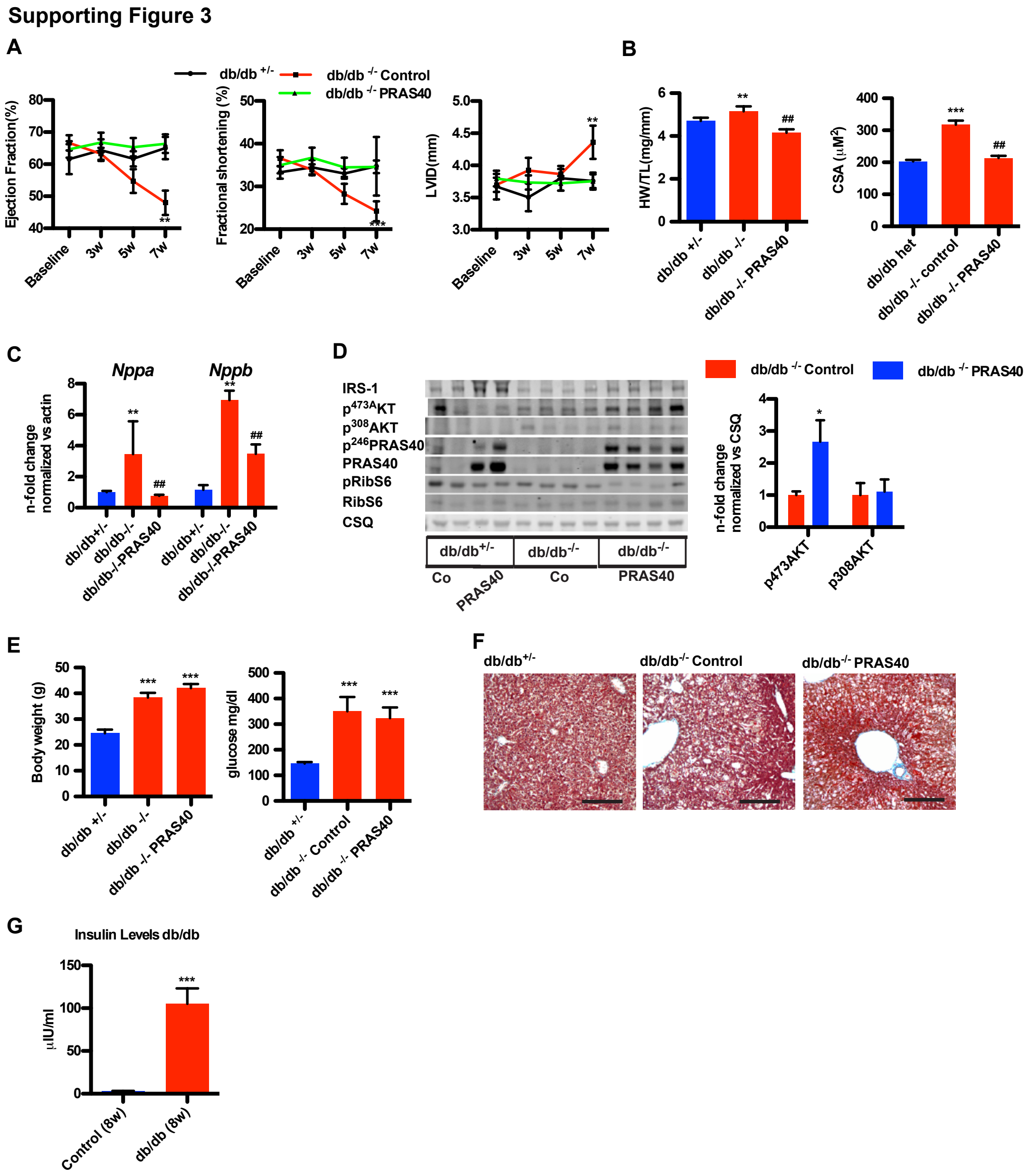

Supporting Figure 3. PRAS40 prevents diabetic cardiomyopathy in db/db mice. **(A)** Line graphs representing echocardiographic assessment of heterozygous control or *db/db* mice for percentage of fractional shortening (FS), ejection fraction (EF) and end-diastolic dimension (LVID), **p<0.01 vs control *db/db*. n=4 per het and 6 per *db/db* group. **(B)** Heart weight to tibia length ratio (HW/TL) in the indicated groups.. (**p<0.01 versus control het; ##p<0.01 versus

control *db/db*). CSA in control and PRAS40 mice (***p<0.01 versus control het; ##p<0.01 versus control *db/db*). **(C)** *Nppa* and *Nppb* levels (**p<0.01 versus control het; ##p<0.01 versus control *db/db*). **(D)** Immunoblots of whole heart lysates. Barplots depicting quantitation of Akt phosporylation. *p<0.05 versus control. **(E)** Body weight and glucose levels in the indicated groups ***p<0.01 vs hets. **(F)** Mason-Trichrome staining of liver sections in the indicated groups. Scale bar 150µm **(G)** Blood insulin levels in the indicated groups. ***p<0.01 vs controls. n=5 in each group.

Suppporting Figure 4

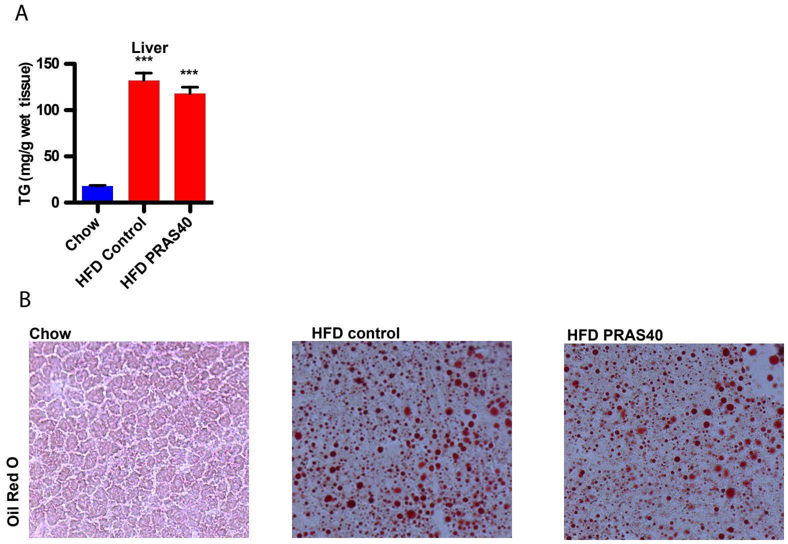

Supporting Figure 4. **(A)** TG content in the liver ***p<0.01 versus control chow. **(B)** Lipid accumulation evidenced by Oil Red O staining. Scale bar 150µm.

Supporting Figure 5

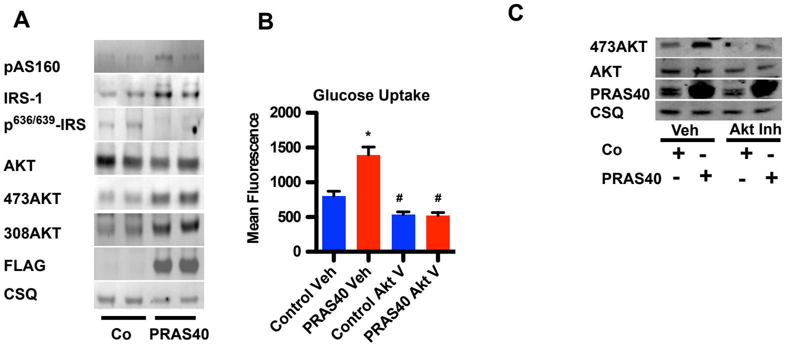

Supporting Figure 5. **(A)** IRS-1 degradation is prevented and Akt phosphorylation is
