# Supplementary material for: PRAS40 prevents development of diabetic cardiomyopathy and improves hepatic insulin sensitivity in obesity
Source: EMBO Mol Med. 2013 Oct 31;6(1):57–65. doi: 10.1002/emmm.201303183 (PMC3936489; doi:10.1002/emmm.201303183)
Supplement: Supplementary file 5 [file emmm0006-0057-sd5.pdf]

control *db/db*). CSA in control and PRAS40 mice (***p<0.01 versus control het; ##p<0.01 versus control *db/db*). **(C)** *Nppa* and *Nppb* levels (**p<0.01 versus control het; ##p<0.01 versus control *db/db*). **(D)** Immunoblots of whole heart lysates. Barplots depicting quantitation of Akt phosporylation. *p<0.05 versus control. **(E)** Body weight and glucose levels in the indicated groups ***p<0.01 vs hets. **(F)** Mason-Trichrome staining of liver sections in the indicated groups. Scale bar 150μm **(G)** Blood insulin levels in the indicated groups. ***p<0.01 vs controls. n=5 in each group.

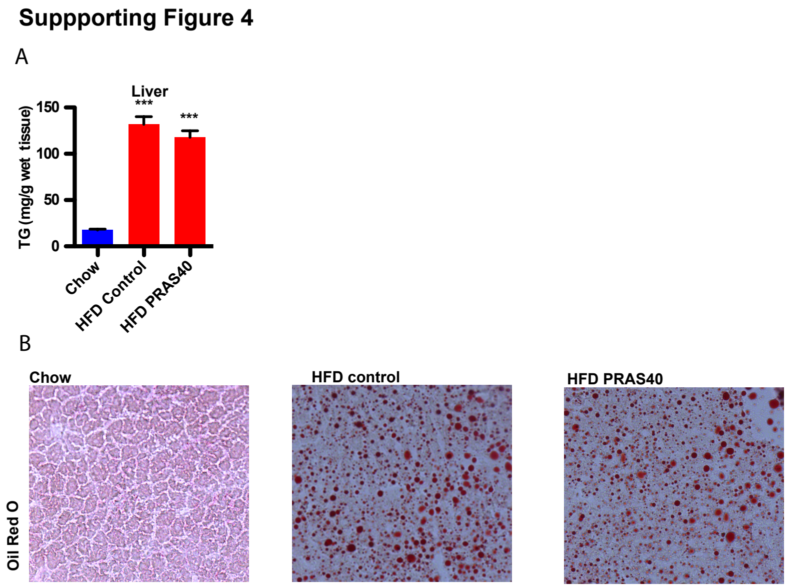

Supporting Figure 4. **(A)** TG content in the liver ***p<0.01 versus control chow. **(B)** Lipid accumulation evidenced by Oil Red O staining. Scale bar 150μm.

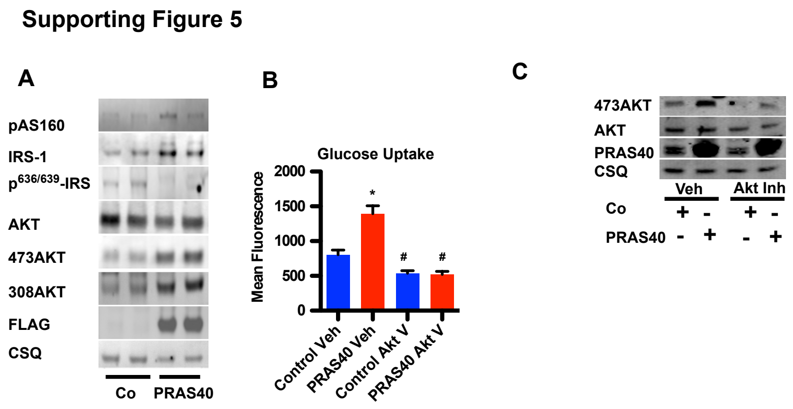

Supporting Figure 5. **(A)** IRS-1 degradation is prevented and Akt phosphorylation is
