# Supplementary material for: PRAS40 prevents development of diabetic cardiomyopathy and improves hepatic insulin sensitivity in obesity
Source: EMBO Mol Med. 2013 Oct 31;6(1):57–65. doi: 10.1002/emmm.201303183 (PMC3936489; doi:10.1002/emmm.201303183)
Supplement: Supplementary file 6 [file emmm0006-0057-sd6.pdf]

control *db/db*). CSA in control and PRAS40 mice (***p<0.01 versus control het; ##p<0.01 versus control *db/db*). **(C)** *Nppa* and *Nppb* levels (**p<0.01 versus control het; ##p<0.01 versus control *db/db*). **(D)** Immunoblots of whole heart lysates. Barplots depicting quantitation of Akt phosporylation. *p<0.05 versus control. **(E)** Body weight and glucose levels in the indicated groups ***p<0.01 vs hets. **(F)** Mason-Trichrome staining of liver sections in the indicated groups. Scale bar 150µm **(G)** Blood insulin levels in the indicated groups. ***p<0.01 vs controls. n=5 in each group.

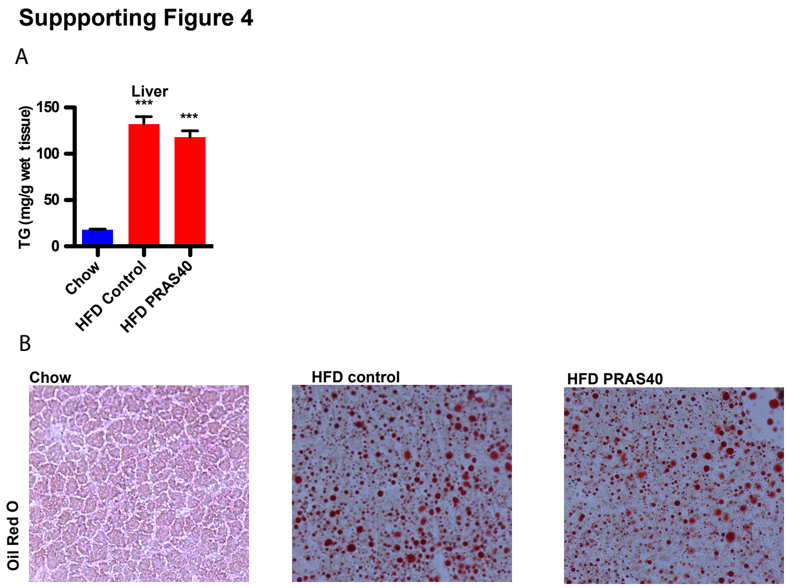

Supporting Figure 4. **(A)** TG content in the liver ***p<0.01 versus control chow. **(B)** Lipid accumulation evidenced by Oil Red O staining. Scale bar 150µm.

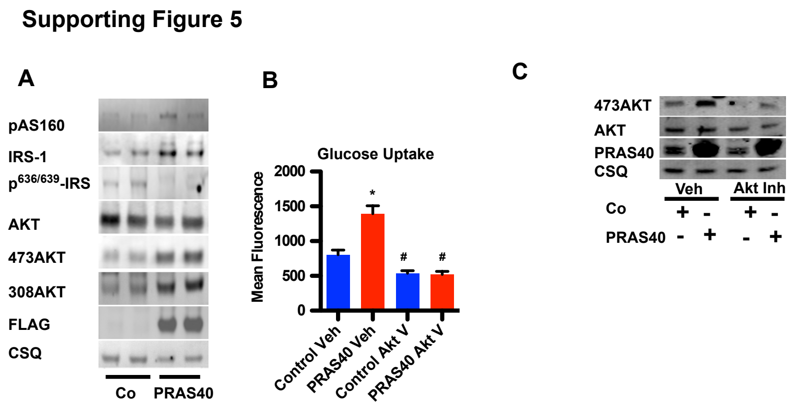

Supporting Figure 5. **(A)** IRS-1 degradation is prevented and Akt phosphorylation is

improved by mTORC1 inhibition with PRAS40**.** **(B)** Glucose uptake measured is increased in PRAS40 NRCMS. Increase in Glucose Uptake is blocked by Akt inhibition (10μM. Akt V Inhibitor) *p<0.05 vs Control. #p<0.05 vs Control. Error bars indicate means ± sem. **(C)** Immunoblot confirming succesful pharmacological Akt inhibition

Supplemental Table 1

**18s Forward**	5’-CGAGCCGCCTGGATACC-3’
**18s Reverse**	5’-CATGGCCTCAGTTCCGAAAA-3’
**ANP Forward**	5’-TGGGTCTTGTTAGGGCTCAAACCT-3’
**ANP Reverse**	5’-TGAAACTCAAGGGACACCCATCGT-3’
**BNP Forward**	5’-AATGGCCCAGAGACAGCTCTTGAA-3’
**BNP Reverse**	5’-CTTGTGCCCAAAGCAGCTTGAGAT-3’
**mPRAS40 Forward**	5’-CGGAGAGCACAGACGACGGC-3’
**mPRAS40 Reverse**	5’-GCACCGACACGGGCAGAGAC-3’
**ATP Synthase Beta 1 For**	CGTGAGGGCAATGATTTATACCAT
**ATP Synthase Beta 1 Rev**	TCCTGGTCTCTGAAGTATTCAGCAA
**Cytochrome C For**	ACCAAATCTCCACGGTCTGTT
**Cytochrome C Rev**	GGATTCTCCAAATACTCCATCAG
**Acadm For**	GGAAATGATCAACAAAAAAAGAAGTATTT
**Acadm Rev**	ATGGCCGCCACATCAGA
**Cpt1b For**	TCTAGGCAATGCCGTTCAC
**Cpt1b Rev**	GAGCACATGGGCACCATAC
**Acadv1 For**	ATCTCTGCCCAGCGACTTT
**Acadv1 Rev**	TTCTGGCTTGTCCAGAACTG
**CPT2 For**	AGTATCTGCAGCACAGCATCGTA
**CPT2 Rev**	GGCTTCTGTGCACTGAGGTATCT
