# Supplementary material for: PRAS40 prevents development of diabetic cardiomyopathy and improves hepatic insulin sensitivity in obesity
Source: EMBO Mol Med. 2013 Oct 31;6(1):57–65. doi: 10.1002/emmm.201303183 (PMC3936489; doi:10.1002/emmm.201303183)
Supplement: Supplementary file 7 [file emmm0006-0057-sd7.pdf]

improved by mTORC1 inhibition with PRAS40. **(B)** Glucose uptake measured is increased in PRAS40 NRCMS. Increase in Glucose Uptake is blocked by Akt inhibition (10µM. Akt V Inhibitor) *p<0.05 vs Control. #p<0.05 vs Control. Error bars indicate means ± sem. **(C)** Immunoblot confirming succesful pharmacological Akt inhibition

Supplemental Table 1

**18s Forward**	5'-CGAGCCGCCTGGATACC-3'
**18s Reverse**	5'-CATGGCCTCAGTTCCGAAAA-3'
**ANP Forward**	5'-TGGGTCTTGTTAGGGCTCAAACCT-3'
**ANP Reverse**	5'-TGAAACTCAAGGGACACCCATCGT-3'
**BNP Forward**	5'-AATGGCCCAGAGACAGCTCTTGAA-3'
**BNP Reverse**	5'-CTTGTGCCCAAAGCAGCTTGAGAT-3'
**mPRAS40 Forward**	5'-CGGAGAGCACAGACGACGGC-3'
**mPRAS40 Reverse**	5'-GCACCGACACGGGCAGAGAC-3'
**ATP Synthase Beta 1 For**	CGTGAGGGCAATGATTTATACCAT
**ATP Synthase Beta 1 Rev**	TCCTGGTCTCTGAAGTATTCAGCAA
**Cytochrome C For**	ACCAAATCTCCACGGTCTGTT
**Cytochrome C Rev**	GGATTCTCCAAATACTCCATCAG
**Acadm For**	GGAAATGATCAACAAAAAAAGAAGTATTT
**Acadm Rev**	ATGGCCGCCACATCAGA
**Cpt1b For**	TCTAGGCAATGCCGTTCAC
**Cpt1b Rev**	GAGCACATGGGCACCATAC
**Acadv1 For**	ATCTCTGCCCAGCGACTTT
**Acadv1 Rev**	TTCTGGCTTGTCCAGAACTG
**CPT2 For**	AGTATCTGCAGCACAGCATCGTA
**CPT2 Rev**	GGCTTCTGTGCACTGAGGTATCT

**HK2 For**	TGCTACAGGTCCGAGCCA
**HK2 Rev**	ATGCTGTCGTCACACGTGC
**PDK4 For**	CCGCTGTCCATGAAGCA
**PDK4 Rev**	GCAGAAAAGCAAAGGACGTT
**Acss2 For**	CTGTGGAGGAGCCACGGGAGTT
**Acss2 Rev**	TGGAGGAATGGGCCAGGGCAT
**PFK For**	CGTTGAGGTAGGAATACTTCTGCA
**PFK Rev**	ACCTCTTCCGAAAGGAGTGGA
**SC5D For**	ccaaatggctggattcatct
**SC5D Rev**	gtccacagggtgaaaagcat
**MVK For**	gggacgatgtcttccttgaa
**MVK Rev**	gaacttggtcagcctgcttc
**UCP3 For**	TTTGGAGCTGGCTTCTGTG
**UCP3 Rev**	AAGGCCCTCTTCAGTTGCTC
**MTE1 For**	GACCTCCCCAAGAGCATAGA
**MTE Rev**	TCCTTGTAGGAGATGGTGTTCC
**IDH2 For**	CCCTATTGCCAGCATCTTTG
**IDH2 Rev**	TGTCCAGGAAGTCTGTGGTG
**PDHA1 For**	GGGACGTCTGTTGAGAGAGC
**PDHA1 Rev**	TGTGTCCATGGTAGCGGTAA
**PFKFB2 For**	CGGGAATGGATCTACACTGG
**PFKFB2 Rev**	GGAGAGCAAAGTGAGGGA TG
**Glut1 For**	GTCCTGCTCGTATTGCTGTG
**Glut1 Rev**	GCCTTTGGTCTCAGGGACTT
**PPARα For**	TCACAAGTGCCTGTCTGTCG
**PPARα Rev**	CAGGTAGGCTTCGTGGATTC
**CD36 For**	GCCAAGCTATTGCGACATGA
**CD 36 Rev**	AAGGCATTGGCTGGAAGAAC

Supplemental Table 2

**Application**	**Antibody**	**Dilution**	**Amplify**	**Company**
**Immunoblot**	Actin	1:2000	no	Santa Cruz (sc-81178)
**Immunoblot**	p246PRAS40	1:1000	no	CST (#2640)
