# Supplementary material for: PRAS40 prevents development of diabetic cardiomyopathy and improves hepatic insulin sensitivity in obesity
Source: EMBO Mol Med. 2013 Oct 31;6(1):57–65. doi: 10.1002/emmm.201303183 (PMC3936489; doi:10.1002/emmm.201303183)
Supplement: Supplementary file 8 [file emmm0006-0057-sd8.pdf]

**HK2 For**	TGCTACAGGTCCGAGCCA
**HK2 Rev**	ATGCTGTCGTCACACGTGC
**PDK4 For**	CCGCTGTCCATGAAGCA
**PDK4 Rev**	GCAGAAAAGCAAAGGACGTT
**Acss2 For**	CTGTGGAGGAGCCACGGGAGTT
**Acss2 Rev**	TGGAGGAATGGGCCAGGGCAT
**PFK For**	CGTTGAGGTAGGAATACTTCTGCA
**PFK Rev**	ACCTCTTCCGAAAGGAGTGGA
**SC5D For**	ccaaatggctggattcatct
**SC5D Rev**	gtccacagggtgaaaagcat
**MVK For**	gggacgatgtcttccttgaa
**MVK Rev**	gaacttggtcagcctgcttc
**UCP3 For**	TTTGGAGCTGGCTTCTGTG
**UCP3 Rev**	AAGGCCCTCTTCAGTTGCTC
**MTE1 For**	GACCTCCCCAAGAGCATAGA
**MTE Rev**	TCCTTGTAGGAGATGGTGTTCC
**IDH2 For**	CCCTATTGCCAGCATCTTTG
**IDH2 Rev**	TGTCCAGGAAGTCTGTGGTG
**PDHA1 For**	GGGACGTCTGTTGAGAGAGC
**PDHA1 Rev**	TGTGTCCATGGTAGCGGTAA
**PFKFB2 For**	CGGGAATGGATCTACACTGG
**PFKFB2 Rev**	GGAGAGCAAAGTGAGGGA TG
**Glut1 For**	GTCCTGCTCGTATTGCTGTG
**Glut1 Rev**	GCCTTTGGTCTCAGGGACTT
**PPARα For**	TCACAAGTGCCTGTCTGTCG
**PPARα Rev**	CAGGTAGGCTTCGTGGATTC
**CD36 For**	GCCAAGCTATTGCGACATGA
**CD 36 Rev**	AAGGCATTGGCTGGAAGAAC

Supplemental Table 2

**Application**	**Antibody**	**Dilution**	**Amplify**	**Company**
**Immunoblot**	Actin	1:2000	no	Santa Cruz (sc-81178)
**Immunoblot**	p246PRAS40	1:1000	no	CST (#2640)

**Immunoblot**	PRAS40	1:1000	no	CST (#2691)
**Immunoblot**	pS6Rib	1:2000	no	CST (#4857)
**Immunoblot**	RibS6	1:500	no	CST (#2317)
**Immunoblot**	p4EBP1	1:1000	no	CST (#2855)
**Immunoblot**	p473AKT	1:1000	no	CST (#4058)
**Immunoblot**	p389S6K	1:500	no	CST (#9234)
**Immunoblot**	IRS-1	1:1000	no	CST (#2390)
**Immunoblot**	p389S6K	1:500	no	CST (#9205)
**Immunoblot**	AKT	1:2000	no	CST (#2966)
**IHC**	α-sarcomeric actin	1:100	no	Sigma (A2172)
**IHC**	PRAS40	1:100	no	CST (#2691)
**IHC**	p473AKT	1:50	yes	CST(#9271)
**IHC**	FLAG	1:100	yes	Sigma (F3165)
