# Supplementary material for: PRAS40 prevents development of diabetic cardiomyopathy and improves hepatic insulin sensitivity in obesity
Source: EMBO Mol Med. 2013 Oct 31;6(1):57–65. doi: 10.1002/emmm.201303183 (PMC3936489; doi:10.1002/emmm.201303183)
Supplement: Supplementary file 9 [file emmm0006-0057-sd9.pdf]

Supplemental Table 3

		Control chow		HFD Chow			
		Control n=5	SEM	Control n=10	SEM	PRAS40 n=10	SEM
LVID (mm)	Baseline	3.67	0.1971	3.6875	0.1727	3.6925	0.09331
	5w	3.653333	0.17221	3.936667	0.082	3.71	0.14799
	10w	3.768333	0.12975	4.165556	0.0936	3.90375	0.06279
	15w	3.744286	0.07913	4.195555	0.048	3.9225	0.04342
	20w	3.82625	0.16506	4.267778	0.0738	4.008889	0.0587
	25w	3.8075	0.22005	4.222	0.0896	3.96	0.06149
LVAW (mm)	Baseline	0.928	0.05748	0.98	0.0503	0.9225	0.0409
	5w	0.978	0.02375	0.92	0.0737	0.888	0.09589
	10w	0.978	0.04663	0.978	0.0534	0.918	0.03087
	15w	1.012	0.04188	1.02	0.0528	0.923	0.04693
	20w	0.963333	0.03283	1.008889	0.0275	1.008889	0.05712
	25w	1.023333	0.08969	1.026	0.0483	0.948	0.05827
FS (%)	Baseline	33.426	2.51014	33.32	1.4703	32.4775	2.19714
	5w	34.538	1.29078	29.29333	1.0351	32.6775	0.73939
	10w	33.15	0.94532	27.415	1.7101	30.74375	1.23782
	15w	34.456	0.74936	25.086	0.904	31.77111	1.63553
	20w	32.99125	1.42874	21.047	0.9831	28.3	0.72822
	25w	33.3925	1.09984	20.915	1.2265	30.212	1.63679
EF (%)	Baseline	58.46666	4.93392	62.555	2.2216	59.806	2.34473
	5w	61.625	2.08043	56.51667	1.644	59.695	2.39117
	10w	60.39286	2.48934	53.132	2.7107	57.04111	2.10859
	15w	64.58111	2.24515	49.861	1.4639	59.91222	2.25469
	20w	62.24222	1.64317	42.858	1.7548	53.842	1.40384
	25w	66.1575	3.24625	42.72	2.087	54.84375	1.84039
E'/A'	10w	1.172083	0.01347	0.666643	0.1349	1.14063	0.13156
