# Supplementary material for: PRAS40 prevents development of diabetic cardiomyopathy and improves hepatic insulin sensitivity in obesity
Source: EMBO Mol Med. 2013 Oct 31;6(1):57–65. doi: 10.1002/emmm.201303183 (PMC3936489; doi:10.1002/emmm.201303183)
Supplement: Supplementary file 10 [file emmm0006-0057-sd10.pdf]

Supporting Information

Table of content:

Supporting Materials and Methods

Supporting Figures 1-5

Supporting Table 1-3

Material and Methods

High fat diet study.

C57Bl/6 were fed a high-fat diet (Calories, Protein 20%, Fat 60%, Carbohydrate 20%) from the age of 7 weeks for 25 weeks in the cardiomyopathy study and for 10 weeks in the liver study.

Echocardiography and Analysis

Echocardiography was used to evaluate cardiac function using a Vevo 770 High-Resolution *In-Vivo* Imaging system. Mice during echocardiographic analysis were subjected to 1-2% isoflourane before assessing cardiac function. End diastolic diameter (LVEDD) and end systolic diameter (LVESD) were measured in order to calculate LV fractional shortening (FS) and LV ejection fraction (EF).

Tissue collection

We sacrificed mice at various time points after removed the hearts and livers. For later isolation of RNA and protein in the study, left ventricles were divided into the left ventricular and right ventricle and stored in in liquid N2.

Retroperfusions

Mice were sacrificed under chloral hydrate sedation. Before the procedure of removing the hearts, mice were weighed in grams. Thereafter, hearts were arrested in diastole by

catheterizing the abdominal aorta and flushing the heart with a high-potassium/cadmium solution. Phosphate buffered formalin fixative was perfused into the coronary arteries at systolic pressure while the left ventricle was filled with formalin at diastolic pressure. Retroperfused hearts were then removed from the chest cavity and placed in formalin for at least 24h, weighed in milligram and processed for paraffin embedding.

Paraffin heart sections were deparaffinized in xylene and rehydrated through graded alcohols to distilled water. Antigen retrieval was achieved by boiling the slides in 10mmol/L citrate pH 6.0 for 12–15 min. Slides were washed several times with distilled water and once with TN buffer (100 mmol/L Tris, 150 mmol/L NaCl). Endogenous tissue peroxidase activity was quenched with TN buffer supplemented with 3% H2O2 for 20 min whenever necessary. Slides were then washed in TN buffer and blocked in TNB buffer (TSATM kit from Perkin-Elmer) at room temperature for at least 30 min. Primary antibodies were applied overnight at 4°C in TNB buffer. The next day, samples were washed in TN buffer and incubated with secondary anti- bodies at room temperature in the dark for 2h. When amplification of signal was needed, slides were washed in TN buffer and incubated with streptavidin horseradish peroxidase-conjugated diluted 1:100 vol/vol in TNB buffer for 30 min at room temperature, and signals were developed using Tyramide substrate diluted 1:50 vol/vol in Amplification Diluent (Perkin-Elmer) for 10 min. Slides were washed in TN buffer and coverslipped using Vectashield eventually in the presence of DNA staining. List of primary and secondary antibodies is reported in Supplemental Table 2.

Immunohistochemistry

Immunostaining of myocytes was performed on cells grown on permanox or glass

chamber slides (gelatin-coated). Cells were fixed by 4% paraformaldehyde (PFA) for 20 min at room tem- perature, permeabilized in PBS supplemented by 0.1% Triton-X for 10 min, and blocked in PBS supplemented with 10% horse serum for at least 30 min. Before starting the protocol and at the end of each successive step, cells were washed in PBS at room temperature. Primary antibodies diluted in blocking solution (PBS with 10% horse serum) were applied overnight at 4 °C. The next day, cells were washed with PBS and incubated for 1h at room temperature and in the dark, with the secondary antibody (Jackson Laboratories) diluted in blocking solution. Conjugated phalloidin 633 (Jackson Laboratories) was diluted in the same buffer at 1:100 vol/vol. Sytox Blue or To-Pro (Molecular Probes) was diluted in Vectashield (Vectra Labs) mounting media at 1:500 vol/vol and used as nuclear staining.

Glucose Tolerance Tests

For glucose tolerance tests (GTT), mice were fasted overnight (16 hr) then administrated 1 g/kg of body weight of glucose by intraperitoneal (i.p.) injection. Blood glucose concentrations were measured before and after the injection at the indicated time points.

Myocyte size measurements

Paraffin sections were stained for tropomyosin to visualize live myocardium wheat germ agglutinin-488 (Carlsbad, Invitrogen) to outline cardiomyocyte membrane and TO-PRO-3 to visualize nuclei. Myocytes were measured using a ImageJ to measure area as μm^3. An n=4 per group was measured.

Cardiomyocyte cell culture and treatment

Neonatal Ventricular cardiomyocytes from 1- to 2d-old rat neonatal hearts (NRCMs) were prepared by trypsin digestion using standard procedures. Cell suspensions were pre-

plated for 2h in M199 medium (Cell-Gro) supplemented with 15% fetal bovine serum (FBS) to reduce non-myocyte cell contamination. Myocytes were plated in gelatin (Sigma) precoated 10cm dishes or in laminin (Sigma) precoated glass slides using the preplating media

Measurement of Glucose Uptake *in vitro*

Glucose uptake by primary cardiomyocytes was analyzed with 2-NBDG (Invitrogen). After 12 hr serum starvation, cells were cultured in 10% DMEM and 200nM NBDG for 6 hr. Cells were washed three times with PBS and fluorescence was measured with Flow cytometry by using a BD FACSAria Flow Cytometer (BD Biosciences).

Measurement of Glucose Uptake *in vivo*

Xenolight RediJect 2-DG 750 (Caliper Life Sciences) was used to measure glucose uptake *in vivo*. NIR fluorescent imaging was performed in extracted hearts and skeletal muscle using the IVIS Spectrum system (Caliper Life Sciences) with a 745-nm excitation and a 820-nm emission filter (1 s acquisition time, F/stop = 2, binning = 4). The mice received i.v. injections of 8 nmol of the 2-DG probes 3h before sacrifice. Total fluorescence signal in tumors (represented by the total radiant efficiency in photons per second per microwatt per square centimeter) were calculated using the Living Image software package (version 4.0, Caliper Life Sciences).

Adeno-associated virus serotype 9 generation and systemic in vivo AAV9 cardiac-targeted gene transfer protocol

For generation of recombinant AAV vector pTRUF-CMVenhMLC800 was constructed by modifying pTRUF12(a gift from R Hajjar) by first removing the region encoding GFP that was down-stream of the IRES. New restriction sites were inserted into the multiple

cloning site to include Nhe1, Pme1, Xho1, and Mlu1. The CMV promoter was replaced with a composite promoter comprised of an 800bp fragment of the MLC2v promoter downstream of a CMV enhancer (a gift from Dr. Oliver J. Muller; see Muller OJ et al. Cardiovasc Res (2006) 70 (1): 70-78). AAV9 vectors with wild-type capsids were generated by cotransfection the helper plasmid pDG-9 (a gift from Dr. Roger Hajjar).

pTRUF-CMVenhMLC800-PRAS40 was created by subcloning the mouse PRAS40 cDNA from pcDNA3.1-PRAS40.

To prepare the recombinant AAV9, HEK293T cells were grown in DMEM/F12 containing 10% FBS, penicillin/streptomycin at 37°C and 5% CO2. HEK293T cells were plated at 8x106 per T-175 flask. Twenty-four hours after plating, cultures were transfected using Polyethylenimine "Max" (MW 40,000; cat. No: 24765; Polysciences, Warrington, PA) as follows 15ug of helper plasmid and 5ug of pTRUF plasmid were mixed with 1 ml of DMEM:F12 and 160ul of polyethylenimine (0.517mg/ml),vortexed for 30 seconds, and incubated for 15 minutes at room temperature. This mixture was then added to the cultures in a drop-wise fashion. The cultures were then rocked intermittently for 15 minutes before incubation. The culture medium was changed 6 to 18hrs later. Three days after transfection, the cells were collected from six flasks and then centrifuged at 500xg for 10 minutes. The cells were resuspended in 10ml of lysis buffer (150 mmol/l NaCl, 50mmol/l Tris-HCL). The resuspended cells were then subjected to three rounds of freeze-thaw followed by treatment with benzonase (1500u of benzonase; Novagen cat. no. 71205) and 1mM MgCl2 at 37°C for 30 minutes. The cell debris was collected by centrifugation at 3,400xg for 20 minutes. The supernatant containing the AAV9 virus was then purified on an iodixanol gradient comprised of the

following four phases: 7.3ml of 15%, 4.9ml of 25%, 4ml of 40%, and 4ml of 60% iodixanol (Optiprep; Sigma-Aldrich, cat. No. D1556) overlayed with 10ml of cell supernatant. The gradients were centrifuged in a 70Ti rotor (Beckman Coulter, Brea, CA) at 69,000 rpm for 1 hour using OptiSeal Polyallomer Tubes (cat. no. 361625; Beckman Coulter). The virus was collected by inserting a needle 2 mm below the 40%-60% interphase and collecting 4 or 5 fractions (~4ml) of this interphase and most of the 40% layer. The fractions were analyzed for viral content and purity by analyzing 10μl of each fraction on a 12% SDS-PAGE gel (BioRad, cat. no. 345-0119) followed by staining with InstantBlue (Expedeon; cat. no. ISB1L) to visualize the viral capsid proteins, VP1, VP2 and VP3. The virus was then collected from the fractions of several gradients and the buffer was exchanged with lactated Ringer's using an ultrafiltration device, Vivaspin 20, 100kDa MWCO (GE Healthcare, cat. no. 28-9323-63). The final viral preparation was then fractionated on a 12% SDS-PAGE gel, stained with InstantBlue, and then compared with a similarly stained gel of a virus of a known titer. Alternatively, a qPCR was performed using a forward primer (AAGTCTCCACCCCATTGACGT) and reverse primer (AGGAGCCTGAGCTTTGATTCC), which spans the CMVenhMLC800 composite promoter. A pTRUF vector containing the CMV/MLC800 promoter was used as a standard to determine copy number.

All viruses are available upon request.

Real-time RT-PCR

Total RNA was isolated from frozen heart or cultured cells by using Quick-RNA™ MiniPrep (Zymo Research) and reverse-transcribed into complementary DNA (cDNA) by using High Capacity cDNA Reverse Transcription Kit (Applied Biosystems). Real-

time PCR was performed on all samples in triplicate using QuantiTect™ SYBR Green PCR Kit (Qiagen) according to the manufacturer's instructions. All primer sequences are shown in Supplemental Table 1 of the Online Data Supplement.

Immunoblotting

Immunoblotting was performed using standard procedures. Protein lysates from ventricles or cultured cardiomyocytes were loaded onto a 4-12% NuPAGE Novex Bis-Tris Gel (Invitrogen) for electrophoresis. Separated proteins were then transferred onto a polyvinylidene fluoride (PVDF) membrane, blocked with 5% skim milk in Tris-Buffered Saline Tween-20 (TBST) for 1h at room temperature, and exposed to primary antibodies. Alkaline phosphatase (AP), horseradish peroxidase (HRP) or Cy5-conjugated IgG (Jackson ImmunoResearch, West Grove, PA) were used as secondary antibodies. Fluorescence signal was detected and quantified by using a Typhoon 9400 fluorescence scanner together with ImageQuant 5.0 software (Amersham Biosciences).
